# Solving UAV 3D Path Planning Based on the Improved Lemur Optimizer Algorithm

**DOI:** 10.3390/biomimetics9110654

**Published:** 2024-10-25

**Authors:** Haijun Liang, Wenhai Hu, Ke Gong, Jie Dai, Lifei Wang

**Affiliations:** Air Traffic Management Institute, Civil Aviation Flight University of China, Deyang 618307, China

**Keywords:** unmanned aircraft, path planning, Lemurs Optimizer algorithm LO, Improved Lemurs Optimizer algorithm ILO, terrain mapping, mathematical model, optimal path

## Abstract

This paper proposes an Improved Lemur Optimization algorithm (ILO), which combines the advantages of the Spider Monkey Optimization algorithm, Simulated Annealing algorithm, and Lemur Optimization algorithm. Through the use of an adaptive nonlinear decrement model, adaptive learning factors, and updated jump rates, the algorithm enhances its global exploration and local exploitation capabilities. A Gaussian function model is used to simulate the mountain environment, and a mathematical model for UAV flight is established based on constraints and objective functions. The fitness function is employed to determine the minimum cost for avoiding obstacles in a designated airspace, and cubic spline interpolation is used to smooth the flight path. The Improved Lemur Optimization algorithm was tested using the CEC2017 benchmark set, assessing its search capability, convergence speed, and accuracy. The simulation results show that ILO generates high-quality, smooth paths with fewer iterations, overcoming the issues of premature convergence and insufficient local search ability in traditional genetic algorithms. It adapts to complex terrain, providing an efficient and reliable solution.

## 1. Introduction

In recent years, unmanned aerial vehicles (UAVs) have demonstrated significant potential across various fields, including agriculture, forestry, high-altitude operations, and wildfire monitoring, owing to their remarkable flexibility and maneuverability [1,2,3,4]. Path planning, as a core technology, plays a pivotal role in determining the effectiveness of UAVs in these applications. However, traditional heuristic methods for path planning in complex three-dimensional environments often exhibit slow performance and lack precision, making it challenging to generate rapid and accurate collision-free paths. Consequently, intelligent optimization-based path planning algorithms have garnered increasing attention for their ability to address these challenges. Numerous intelligent optimization algorithms have been applied to UAV path planning. For instance, the Simulated Annealing algorithm [5] utilizes a simulated annealing probability to determine whether to accept a solution, thereby avoiding entrapment in local optima. However, it is inherently unsuitable for high-dimensional, complex problems. With identical parameter settings, the solutions it generates can vary with each run, complicating the consistent guarantee of finding the global optimum. Next, we consider physics-based algorithms. The Kepler Optimization algorithm (KOA) [6] has yielded promising results in two specific applications; however, its performance is hindered by delayed convergence, which is a significant drawback. Conversely, the electromagnetism-like mechanism (EM) [7] exhibits several limitations, including a tendency to become trapped in local optima, low search efficiency, sensitivity to parameter changes, restricted local search capabilities, and high computational complexity. These issues are further exacerbated in complex, high-dimensional problems, where the EM algorithm may demonstrate less stability than other methods, often necessitating enhancements or hybridization with alternative approaches. The primary weaknesses of the Multi-Verse Optimizer (MVO) [8] include its propensity to become trapped in local optima, inefficient search capabilities, and limited convergence accuracy. Similarly, the Sine Cosine algorithm (SCA) [9] struggles with periodicity in its search process, making it vulnerable to local optima and reducing its ability to adapt dynamically during the search. In general, physics-based algorithms often face high-dimensional constraints in UAV path planning, leading to their susceptibility to local optima. As noted in the literature [10], the Particle Swarm Optimization (PSO) [11] algorithm, when combined with higher-order Bezier curves, produces smoother UAV path planning that aligns better with the UAV kinematic model. However, PSO’s inherent tendency to prematurely converge to local optima remains unresolved. Among population-based algorithms, several noteworthy methods have emerged in recent years, Spider Wasp Optimization (SWO) [12], Crayfish Optimization algorithm (COA) [13], Grey Wolf Optimization (GWO) [14], Glowworm Swarm Optimization [15], and Whale Optimization algorithm (WOA) [16]. Although SWO has some advantages in UAV path planning, such as efficient global search capability and strong convergence performance, it simulates relatively complex biological behaviors, leading to high computational complexity. When dealing with high-dimensional path planning problems, it may consume a large amount of computational resources, which is unfavorable for resource-constrained embedded UAV systems. Despite its overall performance, the Crayfish Algorithm lacks strong exploration capabilities, often leading to local optima in the later optimization stages. Spider Wasp Optimization exhibits strong exploration, exploitation, local optima avoidance, and convergence speed across multiple benchmark tests; however, its sensitivity to parameter settings limits broader applications. In GWO, search efficiency and exploitation heavily rely on parameter α, and improper tuning of this parameter can diminish search performance. Additionally, GWO’s reliance on the alpha wolf’s leadership often reduces population diversity, leading to premature convergence and weaker global search capabilities. The Glowworm Swarm Optimization primarily depends on local interactions between individuals, and as the population approaches the global optimum, these interactions weaken, resulting in poor local search performance and difficulty in finding more precise solutions. Similarly, while the Whale Optimization Algorithm demonstrates decent global search abilities, it struggles during the early stages due to a lack of variance among individuals, hindering exploration. Moreover, WOA’s “encircling” behavior frequently leads to premature convergence on local optima, particularly in multi-modal optimization problems, where individuals tend to cluster, limiting the exploration of other regions within the solution space. A more recent optimization method, the Gold Rush Optimizer (GRO) [17], has garnered attention; however, its application and validation remain limited. Inspired by the behavior of gold seekers, the algorithm’s robustness and efficacy across various complex problems have yet to be fully proven. Simulations of the GRO algorithm applied to UAV path planning revealed that, like other swarm intelligence algorithms, when individual behaviors become overly homogeneous, premature convergence to local optima restricts effective global exploration. To address the challenges of UAV path planning in multi-peak, complex terrains, this paper introduces the Lemur Optimization algorithm (LO) [18]. The LO’s efficiency was validated using the CEC 2011 benchmark set [19], and Its competitiveness in structural engineering problems, such as Transmission Network Expansion Planning (TNEP) and the optimal control of dual-function catalyst mixing, has been demonstrated effectively. With increasing interest in complex 3D UAV path planning, the LO algorithm was applied to this domain. However, simulation results indicated areas for improvement, specifically in search efficiency, local optimization, iteration speed, and total iteration count. In response, this study presents an Improved Lemur Optimization algorithm (ILO), which integrates elements of the Spider Monkey Optimization algorithm, Simulated Annealing, and LO algorithms. The ILO enhances path optimization by adjusting the jump rate and incorporates an adaptive nonlinear decrement model for improved global exploration in early stages and enhanced local exploitation in later stages. Additionally, an adaptive learning factor is employed to dynamically adjust step size and search direction, boosting exploration and accelerating convergence. Testing against the CEC 2017 benchmark set demonstrated that the ILO significantly outperforms the original LO algorithm, delivering greater robustness, improved iteration stability, and fewer required iterations.

## 2. Lemurs Optimizer (LO)

The Lemurs Optimizer (LO) is a metaheuristic optimization algorithm designed to solve global optimization problems. The LO algorithm mimics the behavior of lemurs as they search for food or resources in their environment to enhance search efficiency and optimization performance. It updates the positions of individuals in each generation, using jumping rates and the nearest optimal solution to balance global exploration with local exploitation.

**Figure 1:**
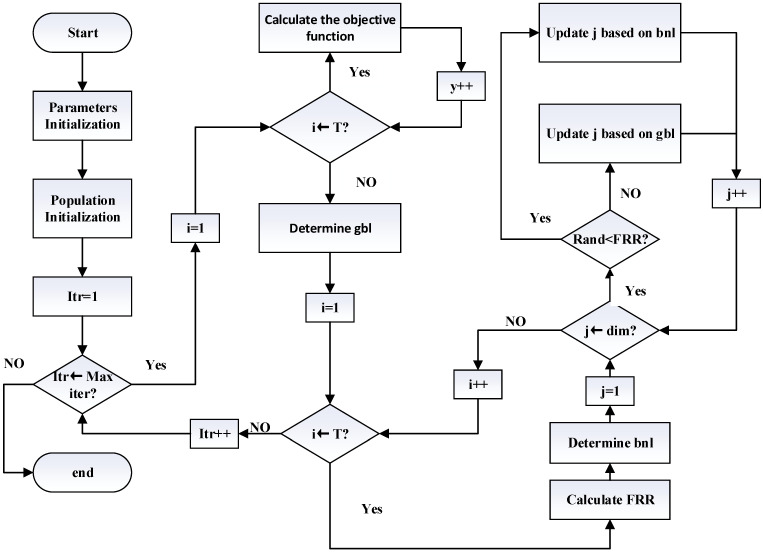
Lemur Optimizer flow chart.

The Lemur Optimizer (LO) algorithm follows several key steps to solve global optimization problems, as illustrated in Figure 1. First, a set of solutions (lemurs) is randomly initialized within the given boundaries. Each lemur’s fitness is evaluated using the objective function. The algorithm then enters a loop for a specified number of iterations. In each iteration, a “jumping rate” is calculated, which gradually decreases over time to balance exploration and exploitation. Based on this jumping rate, each lemur either updates its position by moving toward a neighboring solution or by approaching the best solution found so far. This movement process is influenced by random factors to maintain diversity in the search. Each new solution is evaluated, and if it shows improvement, the lemur‘s position is updated. Throughout the iterations, the algorithm tracks the best solution, updating and displaying results accordingly. The goal is to efficiently explore the search space and converge to the global optimal solution by simulating the natural behavior of lemurs. The specific algorithm structure can be found in Algorithm 1.

The input parameters for the Lemurs Optimizer (LO) algorithm include the following key components: the population size or the number of lemurs, the maximum number of iterations to control the optimization process, the lower and upper bounds for each decision variable that define the search space, the problem’s dimensionality, and the objective function used to evaluate the fitness of each solution. The output of the algorithm includes the best fitness value found during the optimization process, the position vector of the best solution, and a record of the best fitness value at each iteration, providing insight into the algorithm’s convergence behavior.

The goal of the algorithm is to find the global optimal solution to a given problem by simulating the behavior of lemurs. Specifically, the objective function evaluates the position of each individual in every generation, and the algorithm optimizes the objective function value by adjusting these positions. The aim is to find a solution that results in either a minimum or maximum objective value, thereby solving complex global optimization problems effectively.

The set of lemurs is represented in a matrix since the LO algorithm is a population-based algorithm. To do this, the following procedures are carried out. Assuming that we have the population defined as the following matrix:

(1) $$T=\left[\begin{array}{ccc} x_{1}^{1} & \cdots & x_{1}^{d}\\ \vdots & \ddots & \vdots \\ x_{s}^{1} & \cdots & x_{s}^{d} \end{array}\right]$$

In this context, *T* represents the set of lemurs in a population matrix of size s×d, where *d* represents the decision variables, and *s* represents the candidate solutions.

Typically, the decision variable *j* in solution *i* is randomly generated as follows:

(2) $$x_{i}^{j}=xb_{j}+rand()\times \left(ub_{j}-xb_{j}\right)\;\;\forall i\in (1,2,\ldots ,n)\wedge \forall j\in (1,2,\ldots ,d)$$

In Equation (2), the function rand() generates random numbers within a specified range, and the discrete upper and lower bounds of the variable *j* (∀j∈(1,2,…,d)) are represented by $\left[xb_{j},ub_{j}\right]$.

Use the Formula (4) to calculate the free risk rate (*FRR*) and update the globally optimal lemur. For each lemur (identified by index *i*), perform the following steps:

The lemur that has a lower fitness value tends to change its decision variables from the lemur that has a higher fitness value. Lemurs are organized based on their fitness values in each iteration, with one chosen as the global best lemur (denoted as gbl) and one chosen as the best nearest lemur for each lemur (denoted as bnl). In summary, the concept of bnl involves population sorting and selection: In each iteration, the algorithm first sorts the population based on fitness values, evaluating and ranking each lemur from best to worst. Then, the nearest solution is selected: For each individual i, the nearest solution is identified based on its position in the sorted list. This nearest solution, found through the sorted indexes, is closer to the current individual in the search space. Next, the position of the current individual is updated: The individual adjusts its position by calculating the difference between its current solution and the nearest solution, with a random perturbation added to generate a new position. This can be seen as the lemur learning from a better nearby companion to improve its solution. Finally, the update mechanism: If the new solution is better than the current one, the individual’s position is updated; otherwise, the original position is retained.

For each decision variable in lemur *i* (identified by index *j*), assign a random number from [0,1] to rand. If *rand < FRR*, then use the following for:

(3) $$L_{i}^{j}=\left\{\begin{array}{l} x_{i}^{j}+abs(x_{i}^{j}-x_{bnl}^{j})*(rand-0.5)*2;\;rand<FRR\\ x_{i}^{j}+abs(x_{i}^{j}-x_{gbl}^{j})*(rand-0.5)*2;\;rand>FRR \end{array}\right.$$

Update *j* based on the nearest best lemur (bnl). Otherwise, update *j* based on the global best lemur (gbl). Finally, return the globally optimal lemur. In Formula (3), *j* represents the current value of the lemur, $x_{bnl}^{j}$ represents the value of the *j*-th dimension for the best nearest lemur, and $x_{gbl}^{j}$ represents the value of the global best lemur.

Free risk rate (FRR) [20] indicates the risk rate of the all lemurs in the troops, and *rand* represents random numbers between [0,1]. Based on this formulation, it can be concluded that the probability of the FRR is the main coefficient of the LO algorithm. The formula of this coefficient is given in:

(4) $$FRR=FRR\left(High\;Risk\;Rate\right)-CurrIter\times ((High\;Risk\;Rate-Low\;Risk\;Rate)/MaxIter)$$

where Low Risk Rate and High Risk Rate represent constant pre-defined values, MaxIter is the maximum iterations’ number, and CurrIter denotes current iteration. The formula describes how FRR gradually decreases from a high risk to a low risk. It follows a linear decay process, starting from High Risk Rate and gradually reducing to Low Risk Rate as the number of iterations increases.

The CurrIter×((High Risk Rate−Low Risk Rate)/MaxIter represents the part that is reduced in each iteration, ensuring that by the time the maximum iteration count is reached, the FRR value will have decreased to Low Risk Rate.
**Algorithm 1.** The LO algorithm’s pseudocode1:Set up the LO parameters (Number of iterations, Number of dimensions (Dim), Number of solutions, Lower Bound (LB), Upper Bound (UB), Low-Risk Rate, High-Risk Rate, Max iter).2:Generate Lemurs population.3:While the current iteration does not equal the number of iterations do4:Evaluate the objective function for all Lemurs.5:Calculate free risk rate (FRR) using Equation (4).6:Update the Global Best Lemur (gbl).7:For each lemur indexed by i do.8:Update the Best Nearest Lemur (bnl).9:For each decision variable in Lemur i indexed by j do10:Set random ([0, 1]) to rand.11:If rand < Jumping rate then.12:Use Equation (3) case number one to update the decision variable j.13:Else14:Use Equation (3) case number two to update the decision variable j.15:end if16:end for17:end for18:end while19:Return the Global Best Lemur.

## 3. Improved Lemur Optimizer (ILO)

### 3.1. Introduction of Updated Jump Rates

By introducing an innovation in the algorithm concerning the jumping rate within the FRR, the jumping rate decreases as the number of iterations increases. The formula is as follows:

(5) $$JR=initial\;jumping\;rate\times \exp\left(-\frac{iter}{\left(\frac{Max\;iter}{\log\left(\frac{jumping\;rate\;\min}{jumping\;rate\;\max}\right)}\right)}\right)$$

where initial jumping rate is the initial jumping rate, iter is the current number of iterations, and Max iter is the maximum number of iterations, where the constants are $\frac{Max\;iter}{log\left(\frac{jumping\;rate\;min}{jumping\;rate\;max}\right)}$, which controls the rate of decay, where $log\left(\frac{jumping\;rate\;min}{jumping\;rate\;max}\right)$ is a negative value.

Because jumping rate min<jumping rate max, the range of jump rates JR will gradually decrease from the initial value to near the minimum jump rate. The purpose of this design is to allow larger jumps to explore the search space at the beginning of the optimizer process, while gradually decreasing the jump rate at later stages for finer search and local optimizer.

### 3.2. Fusion Spider Wasp Optimizer

The adjustment of the jumping rate incorporates the behavior weight for hunting and nesting (TR).

Definition of TR: Behavior Weight TR is a quadratic increasing function: The value of *TR* increases gradually in a quadratic manner as the number of iterations increases. And mathematical expression:

(6) $$TR={\left(\frac{Current\;Iteration\;Number}{Maximum\;Iteration\;Number}\right)}^{2}$$

Here, TR ranges between 0 and 1.

This behavior weight is a quadratic increasing function, gradually strengthening a certain behavior as the number of iterations increases, adapting to the need for different strategies during the convergence process.

A random decision is made on whether to use hunting and nesting behavior or mating behavior to update the population position using Formula (7).

Hunting and Nesting Behavior: Individuals explore and exploit the search space based on randomly generated parameters, using Levy flights and crossover strategies to update positions.

Mating Behavior: New solutions are generated through mating, and based on fitness, a decision is made whether to accept the new position.

Additionally, the population size is dynamically adjusted, decreasing as the number of iterations increases to enhance the algorithm’s convergence speed. This adjustment is applied to update the lemur population, where the reduction in size corresponds to a decrease in the number of lemurs. By progressively reducing the population throughout the iterations, the algorithm achieves a better balance between global exploration and local exploitation, ultimately improving overall optimization performance.

(7) $$SW_{i}^{t+1}=Crossover(SW_{i}^{t},SW_{m}^{t},CR)$$

where Crossover denotes the uniform crossover operator applied between the solutions of ${SW}_{i}^{t}$ and ${SW}_{m}^{t}$, and *CR* is the crossover rate. ${SW}_{i}^{t}$ and ${SW}_{m}^{t}$ represent the two carriers of male and female spider wasps. Where the Levy flight mechanism [21] simulates a large step leap in the search process, Equation (8):

(8) $$L=\frac{u}{{\left|v\right|}^{1/\beta }}$$

where *u* and *v* are normally distributed random variables and β is a Levy distribution parameter.

By combining LO and SWO, this hybrid optimization approach possesses both global search capabilities (hunting and nesting behavior from SWO) and local exploitation abilities (jump adjustments based on the best solution and nearest solution from LO).

### 3.3. Adaptive Nonlinear Decreasing Model 

#### 3.3.1. Adaptive Jumping Rate

Dynamically adjust the jump rate based on the adaptive nonlinear decrement model [22], allowing it to decrease more rapidly in the later stages. The exponentially decreasing model from Equation (5) is optimized to derive Equation (9), which reduces the jumping rate and improves the local search in the later stages of the iteration.

(9) $$JR=initial\;jumping\;rate\times \exp\left(-\frac{iter^{2}}{\left(\frac{Max\;iter^{2}}{\log\left(\frac{jumping\;rate\;\min}{jumping\;rate\;\max}\right)}\right)}\right)$$

#### 3.3.2. Adaptive CR 

For the SWO part of the algorithm, the crossover rate CR [23] uses an adaptive nonlinear decreasing model, where the crossover probability decreases as the number of iterations increases, reducing the exploratory behavior of the population. The adaptive crossover probability mathematical formula is:

(10) $$CR(t)=CR(0)\times {\left(1-\frac{iter}{Max\;iter}\right)}^{2}$$

where *iter* is the current iteration number, *Max iter* is the maximum iteration number, and *CR*(0) is the initial crossover rate.

**Figure 2:**
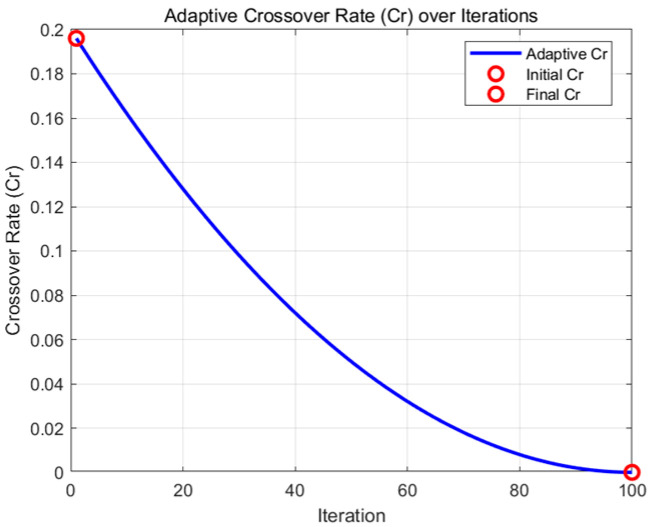
Adaptive cross-probability iteration effect curve.

The trend depicted in Figure 2 is that the crossover probability gradually decreases with the number of iterations, from an initially higher value (approximately 0.2) to a lower value (close to 0). This pattern of change allows the algorithm to explore the solution space by maintaining a greater degree of randomness in the early phases of the algorithm while decreasing randomness in the later phases to allow for more refined development of better solutions.

#### 3.3.3. Adaptive TR 

**Figure 3:**
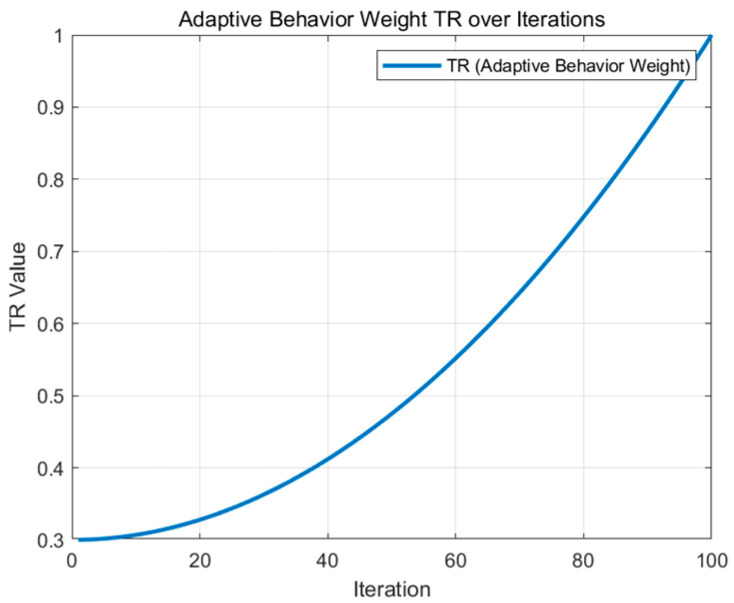
Adaptive behavior weights iteration effect curve.

The setting and adjustment of the adaptive behavior weights help optimize the algorithm’s performance throughout the search process, ensuring that it efficiently finds the optimal solution and adjusts the search strategy appropriately at different stages. The function is formulated as:

(11) $$TR(t)=0.3+0.7\times {\left(\frac{t}{Max\;iter}\right)}^{2}$$

where *t* is the number of contemporary iterations and *Max iter* is the maximum number of iterations. Figure 3 shows a graph of the effect of the adaptive behavior weights TR [24] in iterations, for 100 iterations. The graph illustrates that as the number of iterations increases, the algorithm gradually reduces stochastic exploration during hunting and nesting behavior, and shifts to a more focused local exploitation in order to improve the quality of the solution and eventually converge to the optimal solution.

### 3.4. Combining Improved Simulated Annealing Algorithms 

Combined with the improved Simulated Annealing algorithm, the position update is first performed for each individual *i*. During the position update process, the position update formula for each individual is based on the adaptive jump rate and crossover behavior, which can be expressed as:

(12) $$x_{i}^{\left(t+1\right)}=\left\{\begin{array}{l} x_{i}^{\left(t\right)}+\left|x_{i}^{\left(t\right)}-x_{near}^{\left(t\right)}\right|\cdot (2\cdot rand-1);\;rand<jumping\;rate\\ x_{i}^{\left(t\right)}+\left|x_{i}^{\left(t\right)}-x_{best}^{\left(t\right)}\right|\cdot (2\cdot rand-1);\;rand>jumping\;rate \end{array}\right.$$

In this context, $x_{i}^{\left(t\right)}$ represents the position vector of the *i*-th individual at the *t*-th iteration. $x_{near}^{\left(t\right)}$ represents the position vector of the individual closest to individual *i*. $x_{near}^{\left(t\right)}$ represents the best solution in the current population. Rand is a random number between [0,1]. For each new solution $x_{i}^{\left(t+1\right)}$, if its objective function value $f\left(x_{i}^{\left(t+1\right)}\right)<f(x_{i}^{\left(t\right)})$, the new position is accepted. Otherwise, the probability of accepting the new position follows the simulated annealing acceptance criterion, given by the following probability formula:

(13) $$P=\exp(\frac{-(f(x_{i}^{\left(t+1\right)})-f(x_{i}^{\left(t\right)}))}{T})$$

**Figure 4:**
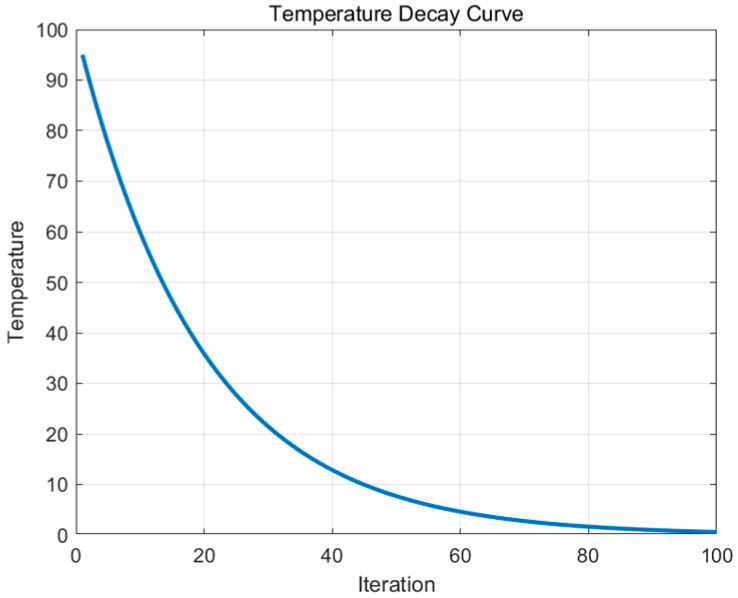
Temperature decay curve.

In this context, *T* represents the current temperature. The acceptance criterion is as follows: If *P* is greater than a random number rand between [0, 1], the new solution is accepted; otherwise, the old solution is retained. Finally, the temperature is updated $T^{\left(t+1\right)}=T_{0}\cdot \alpha^{t}$, where $T_{0}$ is the initial temperature, and α is the cooling factor, typically 0 <α< 1, Figure 4 shows the temperature decay curve after the temperature update. In this curve, the temperature represents the algorithm’s probability of accepting inferior solutions. A higher temperature means the algorithm is more likely to accept worse solutions to avoid becoming stuck in local optima. As the temperature decreases, the algorithm becomes increasingly “greedy”, focusing more on local optima and reducing the acceptance of worse solutions.

### 3.5. Adding Adaptive Learning Factors 

Define the learning factor parameters:

Set the initial learning factor, minimum and maximum learning factors, and their decay rate as shown in Algorithm 2.

Dynamic adjustment of learning factors [25]:

The value of the learning factor is dynamically adjusted according to the number of iterations to influence the search direction and step size update.

**Figure 5:**
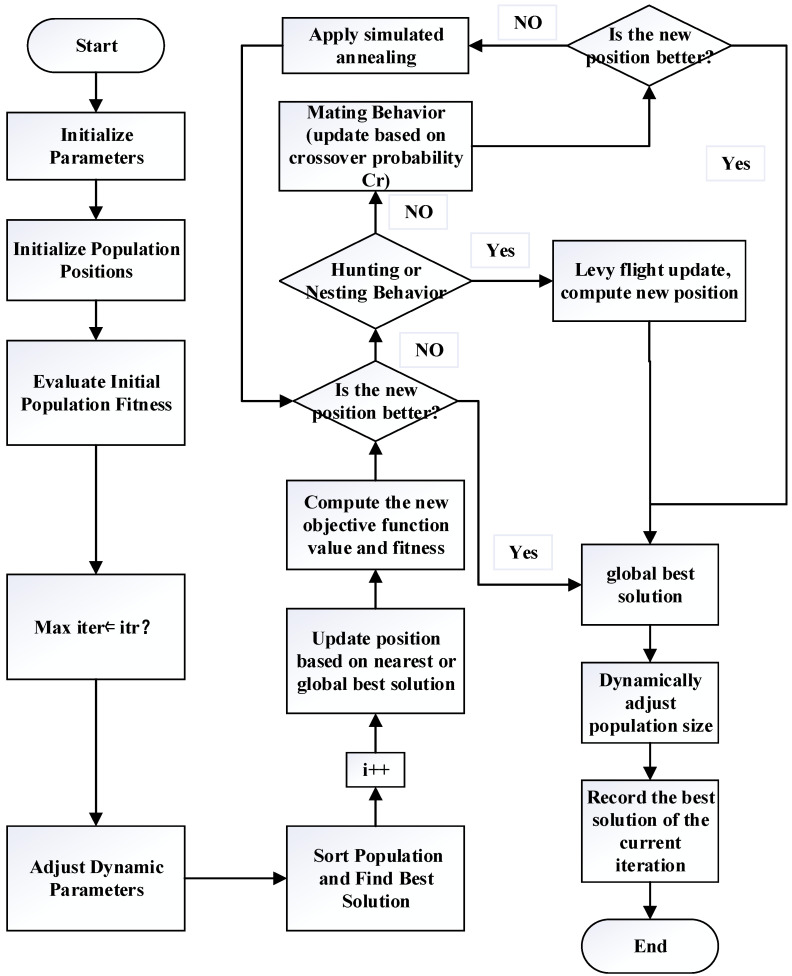
Improved Lemur Optimizer flow chart.

Applying learning factors to algorithms:

Using learning factors to adjust the search direction and step size to enhance search capability and accelerate convergence. The detailed ILO algorithm process is shown in Figure 5.
**Algorithm 2.** The ILO algorithm’s pseudocode1:Set up the ILO parameters (Number of iterations, Number of dimensions (Dim), Number of solutions, initial jumping rate, jumping rate min, jumping rate max, TR, Cr, T0, *α*, initial learning factor, min learning factor, max learning factor, learning factor decay).2:Initialize population positions.3:Evaluate initial population fitness.4:Enter main loop (iteration t from 1 to Max iter).5:Adjust dynamic parameters.6:Sort population and find the current best solution7:For each agent i:8:Select the nearest optimal solution or the global best solution to update the position.9:Update each decision variable j’s position based on jumping rate.10:If random value rand < jumping rate.11:Update position based on the nearest best lemur.12:Else:13:Update position based on the global best lemur.14:Compute the new objective function value and fitness15:If the new position is better16:Update the agent’s position and fitness17:If the new solution is better than the global best, update the global best solution18:Otherwise19:Apply simulated annealing and accept a worse solution with a certain probability20:If rand < TR (hunting/nesting behavior)21:For each agent i.22:Adjust position using Levy flight.23:Compute the new objective function value24:If the new position is better, update the agent’s position and the global best solution25:Otherwise, apply simulated annealing to accept the worse solution with a certain probability26:Else27:For each agent i.28:Update position based on crossover probability Cr.29:Compute the new objective function value30:If the new position is better, update the agent’s position and the global best solution31:Otherwise, apply simulated annealing to accept the worse solution with a certain probability32:Dynamically adjust population size33:Record the best solution of the current iteration34:End

## 4. Simulation Test and Result Analysis of Improved Lemur Optimizer

To validate the performance of the Improved Lemur Optimization algorithm (ILO), it was compared with five other algorithms: Particle Swarm Optimization (PSO), Lemurs Optimizer (LO), Spider Wasp Optimizer (SWO), Kepler Optimization algorithm (KOA), and Gold Rush Optimizer (GRO). The following describes the test functions of the experimental data, comparison algorithms, parameter configurations, and analysis of the experimental data. The experiment was conducted on a Thunderobot laptop from Qingdao, China, equipped with a 12th Gen Intel(R) Core i5-12450H processor with a base frequency of 2.50 GHz and 256 GB of memory, and MATLAB software version R2023b installed.

### 4.1. Comparison of Test Function Results

In this study, we assessed the performance of the ILO algorithm using 30 test functions from the CEC2017 benchmark suite [26], comparing it against seven benchmark algorithms, including GRO, LO, PSO, SWO, and KOA (refer to Table 1 for details). The parameter settings for these algorithms are provided in Table 2. To ensure experimental fairness, all algorithms were initialized with a uniform population size of 30 and a maximum iteration limit of 500, with evaluations conducted for both 30- and 100-dimensional problems. Performance metrics, including the mean, maximum, minimum, standard deviation, and rank-sum test results, were derived from 100 independent runs per function. After 500 iterations, the ILO algorithm demonstrated its superior global search efficiency, consistently achieving the best results across all evaluation criteria, including mean, maximum, minimum, standard deviation, and rank-sum tests. In Table 1, the last line, “Search Range: ${\left[-100,100\right]}^{D}$”, indicates that the search range of all test functions is within the D-dimensional space of [−100,100]. The numbers in the last column represent the benchmark optimal values Fi* for the CEC’17 test functions. These values indicate the function’s value, Fi(x*), at the global optimal solution x*. For example, for the first function (Shifted and Rotated Bent Cigar Function), the optimal function value Fi(x*) is 100 when the global optimal solution is found. Similarly, for other functions, the numbers represent the best possible value of the function at its global optimum. These benchmark values are used to evaluate the performance of optimization algorithms, with the goal being to find solutions that are as close as possible to these optimal values.

### 4.2. Results and Analysis of Cec2017 Benchmark Functions

CEC2017 comprises a set of 30 single-objective benchmark functions, encompassing a diverse range of characteristics: F1 and F2 are unimodal functions, F3 to F9 are basic multimodal functions, F10 to F19 are hybrid functions, and F20 to F30 are composite functions. As illustrated in Table A1 and Table A2 in Appendix A and Appendix B, the results of the ILO algorithm after being executed with dimensions of 50 and 100 reveal the following key performance advantages of the ILO algorithm:

In the 50-dimensional case, although PSO slightly outperforms ILO on F10 and GRO shows a minor advantage on F17 and F20, the ILO algorithm clearly outperforms the remaining functions. In the 100-dimensional scenario, while SWO marginally surpasses ILO on F20, ILO exhibits substantial dominance across all other functions.

In both 50- and 100-dimensional cases, the ILO algorithm consistently performs exceptionally well on unimodal functions F1 and F2, which feature a single global optimum with no local traps. This simplifies the search process by eliminating the need to navigate through complex local optima. Even with higher dimensionality, the ILO algorithm demonstrates remarkable adaptability, striking an effective balance between exploration and exploitation. This balance ensures efficient resource utilization, rapid convergence to the optimal solution, and minimal redundant exploration.

On multimodal functions F3 and F9, the ILO algorithm exhibits strong global exploration capabilities in both 50- and 100-dimensional cases. It effectively avoids getting trapped in local optima, progressively directing its focus toward the optimal solution. As dimensionality increases, the ILO algorithm maintains its scalability and robustness, consistently identifying global or near-optimal solutions in complex landscapes while preserving population diversity. This enables thorough exploration and a well-balanced transition from early-stage exploration to later-stage exploitation.

The ILO algorithm performs exceptionally well across most composite functions (F10 to F30) in both 50- and 100-dimensional cases, especially in terms of maximum, minimum, mean, and standard deviation, highlighting its stability and robustness in managing high-dimensional, complex problems. Although PSO, GRO, and SWO show slight advantages in specific functions (F17, F20), indicating their effectiveness in structured problems, the ILO algorithm consistently demonstrates superior global optimization capabilities. Its scalability and ability to handle increasing dimensional complexity are particularly evident in high-dimensional scenarios.

A *p*-value below 0.05, as outlined in [28], signifies a statistically significant difference, confirming that one algorithm outperforms the other. Conversely, a *p*-value above 0.05 indicates no significant difference, with observed variations likely attributable to randomness. In this study, *p*-value analysis reinforces the ILO algorithm’s superiority, as demonstrated in Table A1 and Table A2 in Appendix A and Appendix B, where most test functions yield *p*-values below 0.05. This validates ILO’s exceptional performance in global search capability, convergence speed, and robustness, particularly in high-dimensional problems. The differences between the ILO algorithm and other algorithms are particularly noticeable in unimodal functions F1–F2 and multimodal functions F3–F9. This highlights the ILO algorithm’s superior performance in handling both simple and complex optimization problems, further underscoring its effectiveness in global search and rapid convergence.

### 4.3. Comparison of Convergence Curves and Box Plots for 50dimCec2017 Benchmark Functions

**Figure 6:**
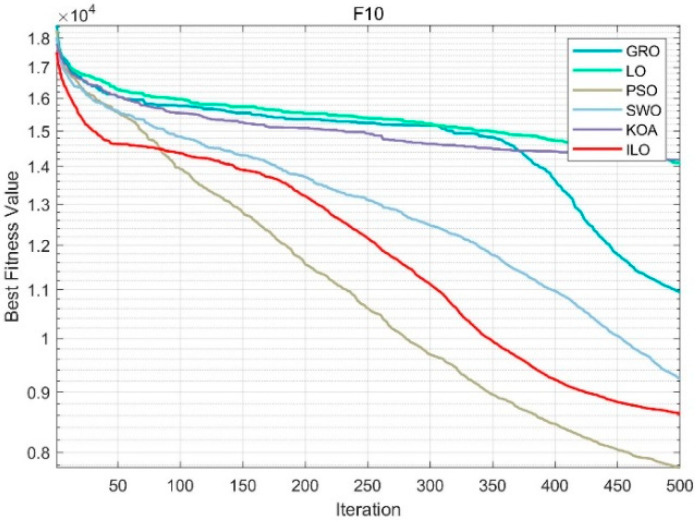
F10 (50dim).

**Figure 7:**
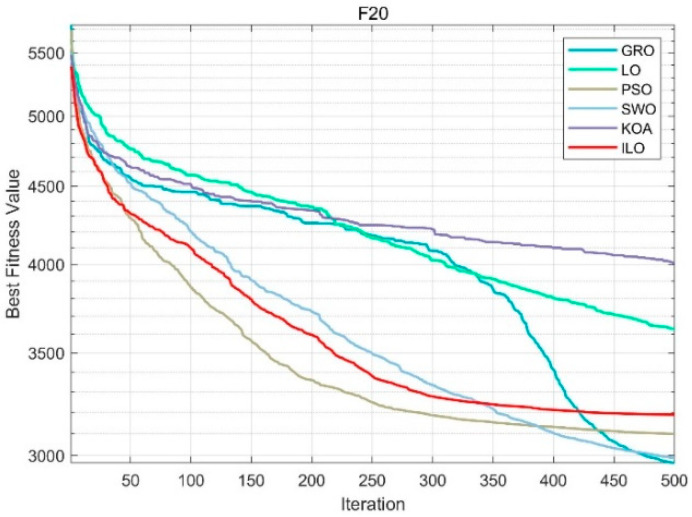
F20 (50dim).

**Figure A1:**
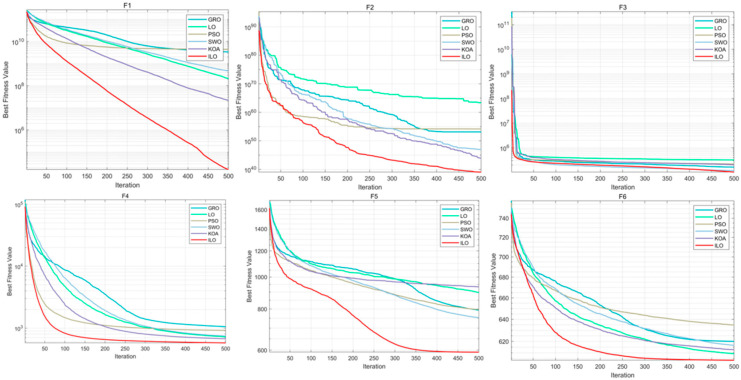
F1–F6 (50dim).

**Figure A2:**
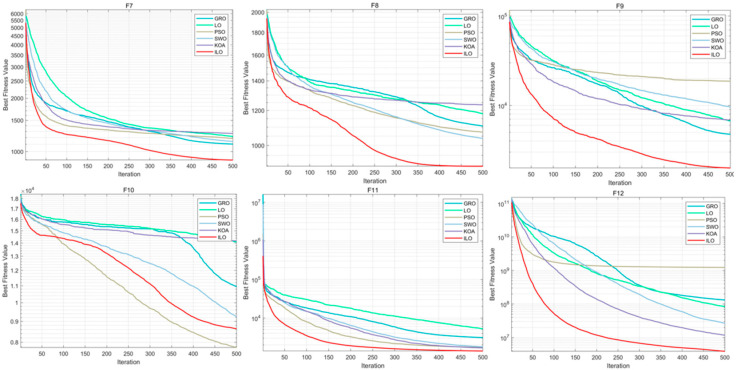
F7–F12 (50dim).

**Figure A3:**
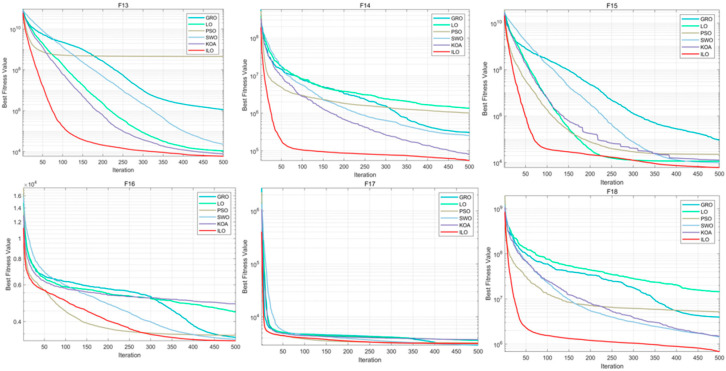
F13–F18 (50dim).

**Figure A4:**
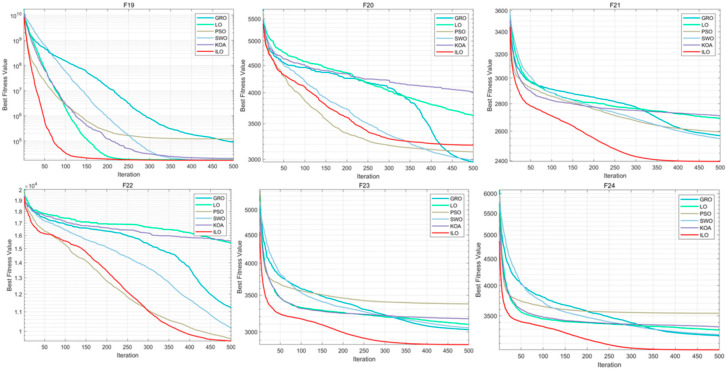
F19–F24 (50dim).

**Figure A5:**
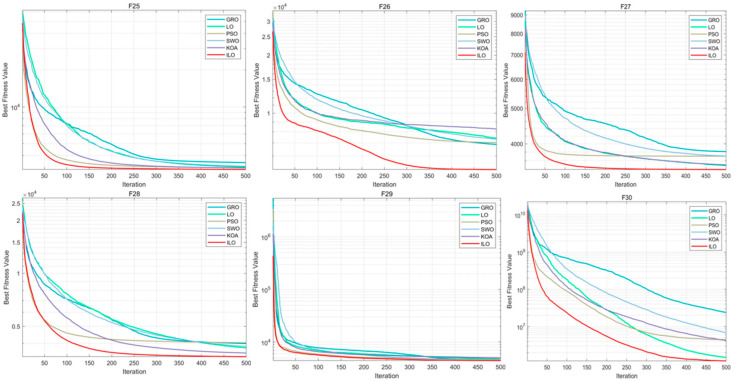
F25–F30 (50dim).

From Figure A1, Figure A2, Figure A3, Figure A4 and Figure A5 in Appendix C, it can be seen that F1 and F2 are unimodal functions, while F3 to F9 are simple multimodal functions. The convergence curves of the ILO test functions indicate that the convergence speed of the ILO method is generally faster than that of PSO, GRO, LO, KOA, and SWO algorithms. This also demonstrates that the ILO algorithm has strong competitiveness in solving both simple and complex problems. From Figure 6, in the case of the F10 function, although the initial speed is slower, it shows strong optimization ability in the middle and later stages, and the final solution is superior to algorithms like PSO and SWO. It is also able to continuously find better solutions even in the later stages. Functions F20 to F30 are 10 high-dimensional composite functions used to evaluate the global exploration capability of the algorithms. Due to not having a global optimum, they are challenging to optimize. From Figure 7, in the F20 function, the ILO algorithm converges quickly in the early stages, and the convergence trend stabilizes in the middle and later stages, avoiding premature convergence. The quality of the final solution is significantly better than that of PSO, SWO, and KOA. Its ability to continuously optimize the solution over a long iteration process demonstrates its strong robustness. From the convergence of functions F11 to F30, it is clear that the ILO algorithm outperforms PSO, GRO, LO, KOA, and SWO in terms of convergence speed and accuracy in 50-dimensional problems. This shows that when optimizing high-dimensional functions, other algorithms tend to fall into local optima, significantly reducing their convergence speed.

**Figure A6:**
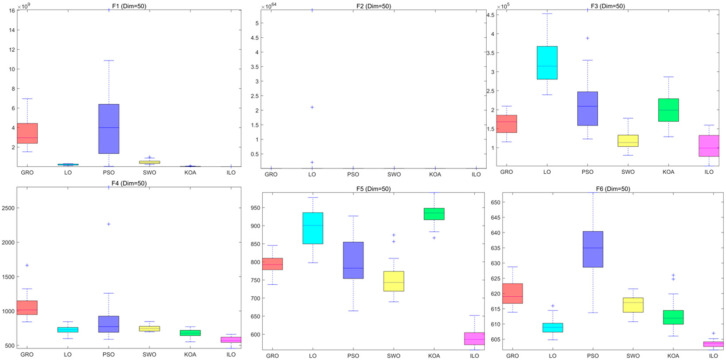
F1–F6 (50dim) box plot (math.).

**Figure A7:**
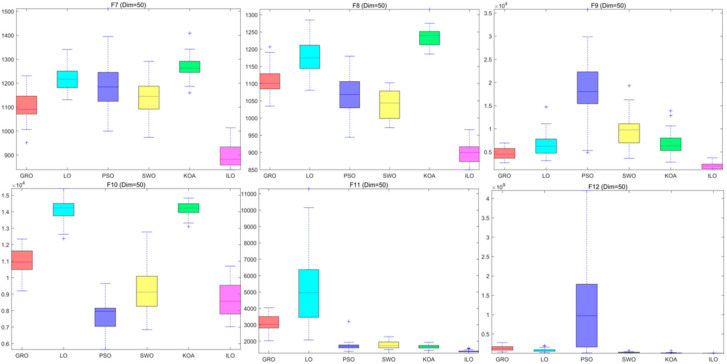
F7–F12 (50dim) box plot (math.).

**Figure A8:**
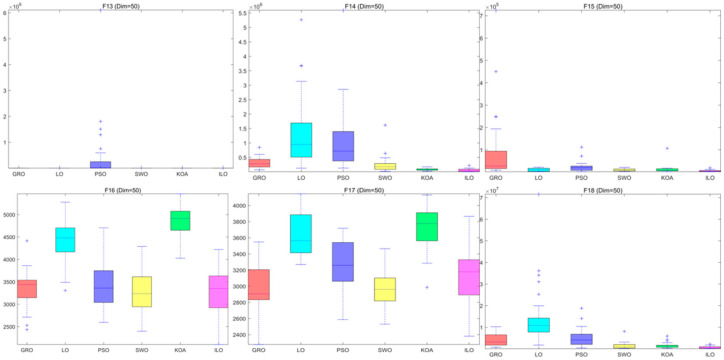
F13–F18 (50dim) box plot (math.).

**Figure A9:**
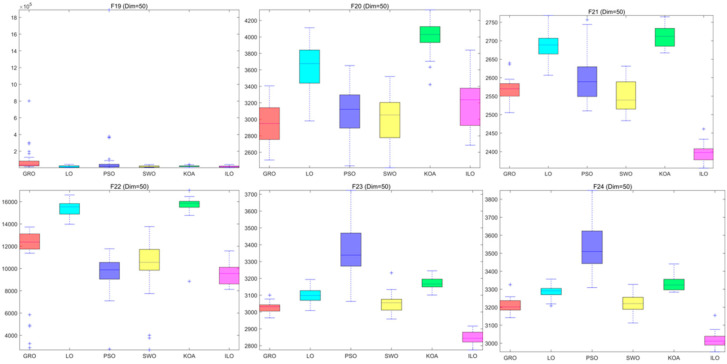
F19–F24 (50dim) box plot (math.).

**Figure A10:**
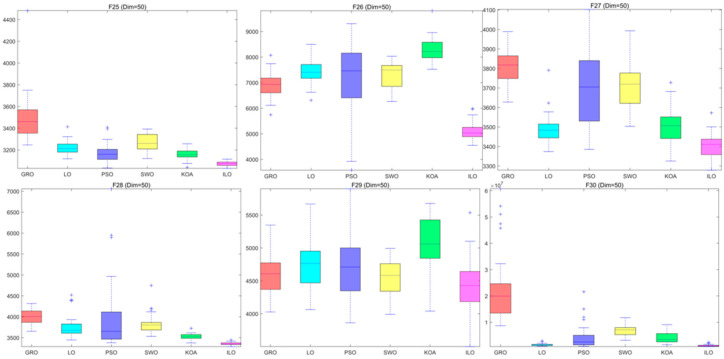
F25–F30 (50dim) box plot (math.).

In Appendix D, the box plots from Figure A6, Figure A7, Figure A8, Figure A9 and Figure A10 represent a simplified version of the curves shown in Figure A1, Figure A2, Figure A3, Figure A4 and Figure A5. The numbers on the left side of the plots indicate the optimal fitness values. In the box plots, the upper dashed line represents the maximum value, and the lower dashed line represents the minimum value. The box itself represents the area where most of the fitness values are concentrated. Therefore, by observing the box plots, one can intuitively determine the optimal performance.

From the box plots in Figure A6, Figure A7, Figure A8, Figure A9 and Figure A10 [29], it can be seen that the ILO algorithm has almost a strong advantage over the PSO, GRO, LO, KOA, and SWO algorithms in terms of mean, maximum, and minimum values.

### 4.4. Comparison of Convergence Curves and Box Plots for 100dimCec2017 Benchmark Functions

**Figure 8:**
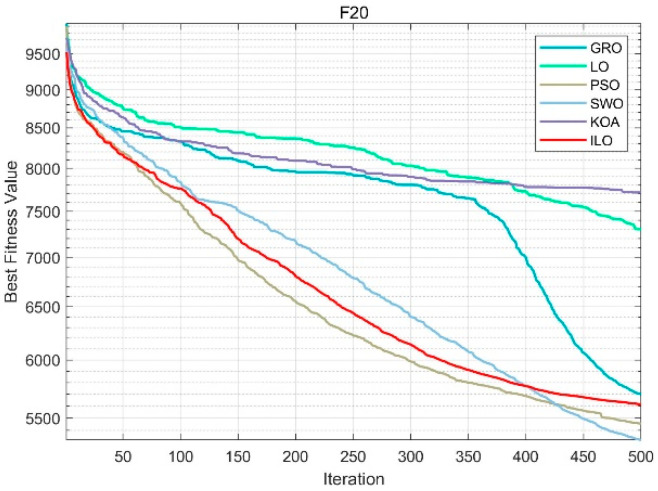
F20 (100dim).

**Figure A11:**
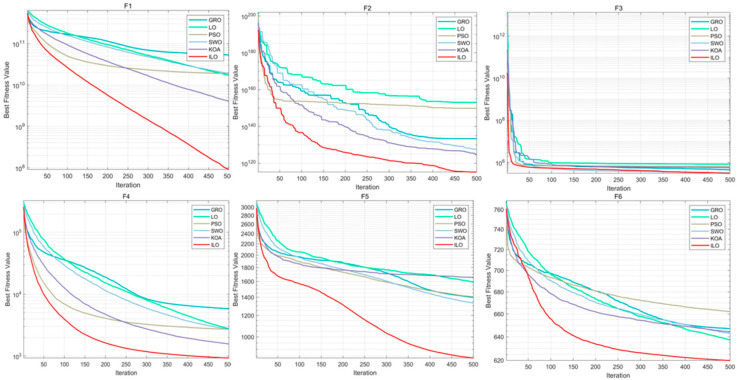
F1–F6 (100dim).

**Figure A12:**
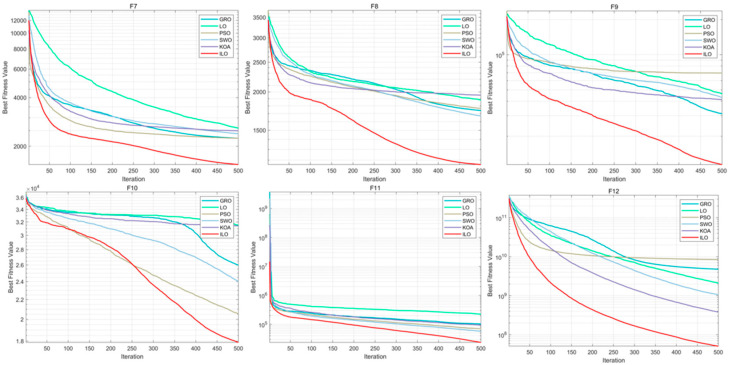
F7–F12 (100dim).

**Figure A13:**
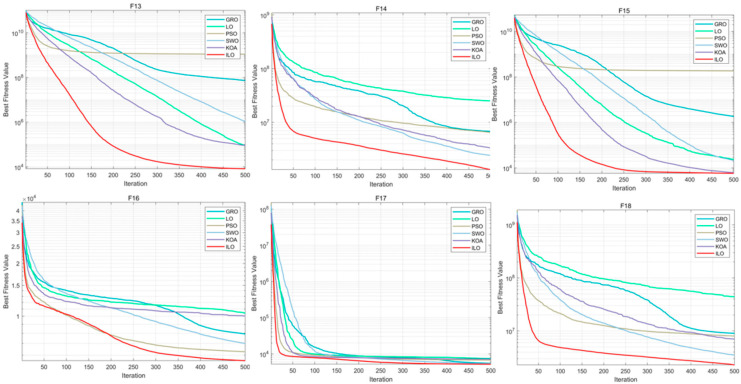
F13–F18 (100dim).

**Figure A14:**
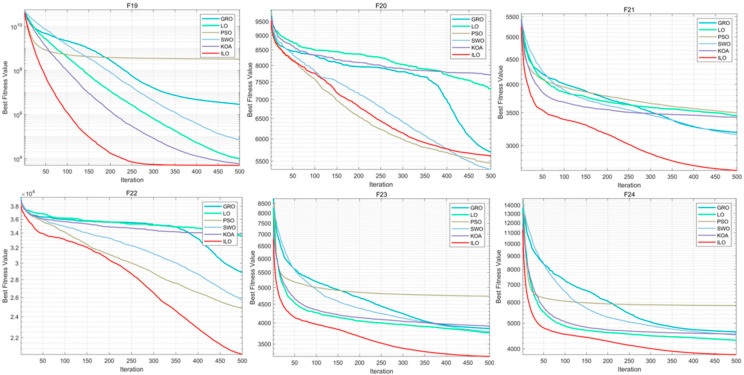
F19–F24 (100dim).

**Figure A15:**
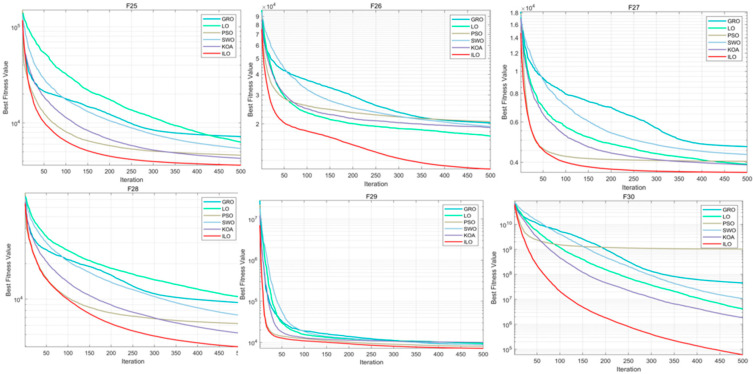
F25–F30 (100dim).

From Figure A11, Figure A12, Figure A13, Figure A14 and Figure A15 in Appendix E, it can be observed that in cases ranging from 50 to 100 dimensions, the performance advantage of the ILO algorithm is significantly enhanced. Whether dealing with simple problems or complex composite problems, it achieves the optimal iterative results more stably and earlier than other algorithms. In Figure 8 for Function F20, the ILO algorithm demonstrates strong global search capabilities in the early stages and continues to improve the solution quality without stagnation in later stages. The final convergence result surpasses other algorithms like KOA, PSO, and SWO.

By comparing the convergence curves of other functions, it is evident that increasing the dimensions from 50 to 100 greatly expands the search space, making the solution space more complex. Many algorithms tend to experience performance degradation or instability in high-dimensional scenarios. However, the ILO algorithm consistently performs well in high dimensions, showcasing its excellent scalability and robustness. As the dimensionality increases, the difficulty of optimization problems usually rises significantly—a phenomenon known as the “curse of dimensionality.” The outstanding performance of the ILO algorithm indicates that it adapts well to this curse. By utilizing improved Simulated Annealing algorithms and Levy flight strategies, it maintains adaptability to high-dimensional problems and excels in addressing complex optimization challenges.

**Figure A16:**
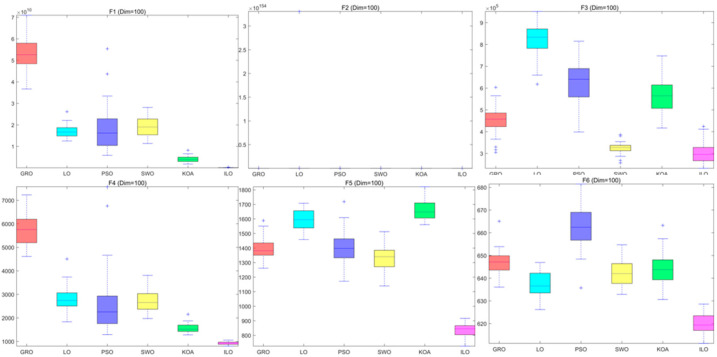
F1–F6 (100dim) box plot (math.).

**Figure A17:**
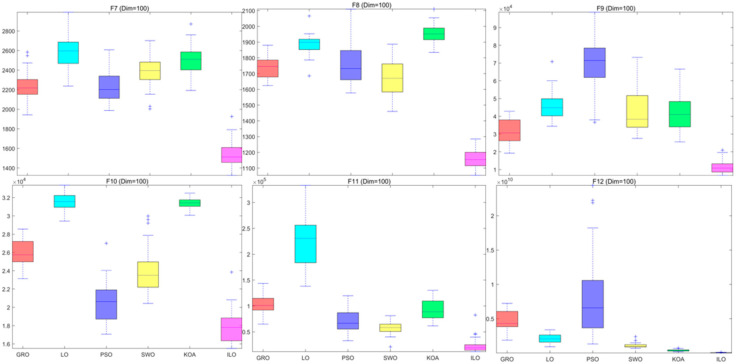
F7–F12 (100dim) box plot (math.).

**Figure A18:**
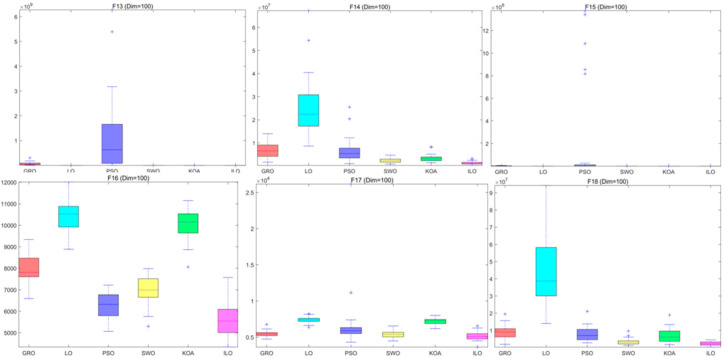
F13–F18 (100dim) box plot (math.).

**Figure A19:**
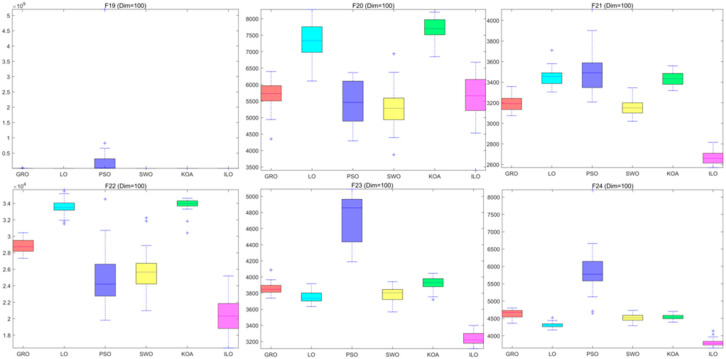
F19–F24 (100dim) box plot (math.).

**Figure A20:**
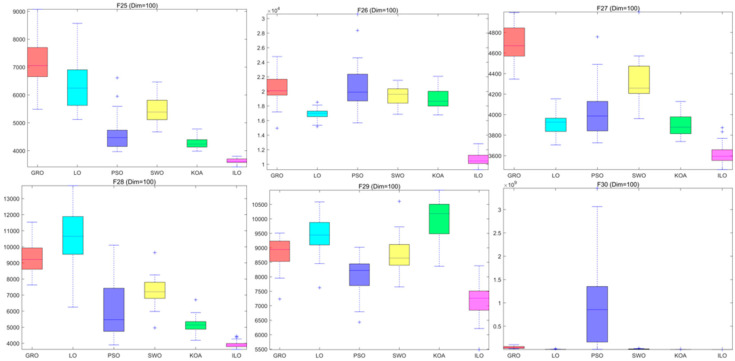
F25–F30 (100dim) box plot (math.).

In Appendix F, the box plots in Figure A16, Figure A17, Figure A18, Figure A19 and Figure A20 represent a simplified version of the curve charts from Figure A11, Figure A12, Figure A13, Figure A14 and Figure A15. They serve as a concise representation of the data from the curve plots. The numbers on the left side of the box plots indicate the optimal fitness values. In each box plot, the highest point of the dashed line denotes the maximum value, while the lowest point of the dashed line indicates the minimum value. The box itself represents the area where most of the fitness values are concentrated. Thus, by examining the box plots, one can intuitively identify the optimal performance.

From the box plots in Figure A16, Figure A17, Figure A18, Figure A19 and Figure A20, it can be seen that the ILO algorithm demonstrates the greatest advantage in terms of mean, maximum, and minimum values compared to the PSO, GRO, LO, KOA, and SWO algorithms in the 100-dimensional case. This indicates that the ILO algorithm performs well across different dimensions, avoiding premature convergence and continuously improving solution quality. It is capable of handling complex high-dimensional problems, while other algorithms may perform poorly in high-dimensional cases. Additionally, the ILO algorithm produces more consistent results across multiple runs, avoiding extreme outcomes.

In summary, the ILO algorithm outperforms other algorithms in both 50-dimensional and 100-dimensional scenarios. The reason for this is that the ILO algorithm initially uses an updated jump rate process to allow for larger jumps to explore the search space. In the later stages, the jump rate gradually decreases to enable finer search and local optimization. The algorithm also incorporates the Spider Monkey Optimization algorithm, which leverages three behaviors to dynamically reduce population size during iterations, thereby improving convergence speed. The Levy flight mechanism facilitates large jumps in the simulated search process. The adaptive nonlinear decreasing model further optimizes the jump rate, reducing the crossover probability in the partially integrated Spider Monkey Optimization algorithm and improving optimization. Additionally, simulated annealing is applied when processing each solution—if the current solution has a poor objective value, the simulated annealing probability determines whether to accept the solution, helping to avoid local optima. The inclusion of an adaptive learning factor strengthens the algorithm’s iteration and search capabilities.

## 5. Comparison of UAV Path Planning Applications

### 5.1. Environmental Modeling

In this study, an accurate 3D environment model is constructed for UAV trajectory planning simulation. The flight area is set as a 100 × 100 × 250 rectangular coordinate space, and the Gaussian function model [30,31] is used to simulate obstacles such as mountain peaks, which can accurately reproduce the terrain’s ups and downs and also flexibly adapt to different geographic environments. The height distribution of each peak can be expressed as a Gaussian function:

(14) $$h_{i}(x,y)=H_{i}\exp\left(-\left(\frac{{\left(x-x_{i}\right)}^{2}}{2\sigma_{x_{i}}^{2}}+\frac{{\left(y-y_{i}\right)}^{2}}{2\sigma_{y_{i}}^{2}}\right)\right)$$

In Equation (14), $h_{i}(x,y)$ represents the height of the i-th peak at position (x,y). $H_{i}$ is the height of the i-th peak, and ($x_{i},y_{i}$) is the center position of the i-th peak. $\sigma_{x_{i}}$ and $\sigma_{y_{i}}$ are the standard deviations of the i-th peak in the *x* and *y* directions, respectively, determining the extent of the peak’s spread. The total terrain height is the sum of the heights of all peaks:

(15) $$z(x,y)=\overset{N}{\underset{i=1}{\sum }}h_{i}(x,y)=\overset{N}{\underset{i=1}{\sum }}H_{i}\exp\left(-\left(\frac{{\left(x-x_{i}\right)}^{2}}{2\sigma_{x_{i}}^{2}}+\frac{{\left(y-y_{i}\right)}^{2}}{2\sigma_{y_{i}}^{2}}\right)\right)$$

In Equation (15), N is the total number of peaks, and z(x,y) represents the terrain height at the horizontal coordinates (x,y). To generate these peaks, we randomly determine the center position, height, and range of each peak:

1. ($x_{i},y_{i}$) is the center position of the i-th peak within the map’s boundaries.

2. $H_{i}$ is the height of the i-th peak.

3. $\sigma_{x_{i}},\sigma_{y_{i}}$ control the slope by adjusting the rate of change of the peak along the *x* and *y* axes.

**Figure 9:**
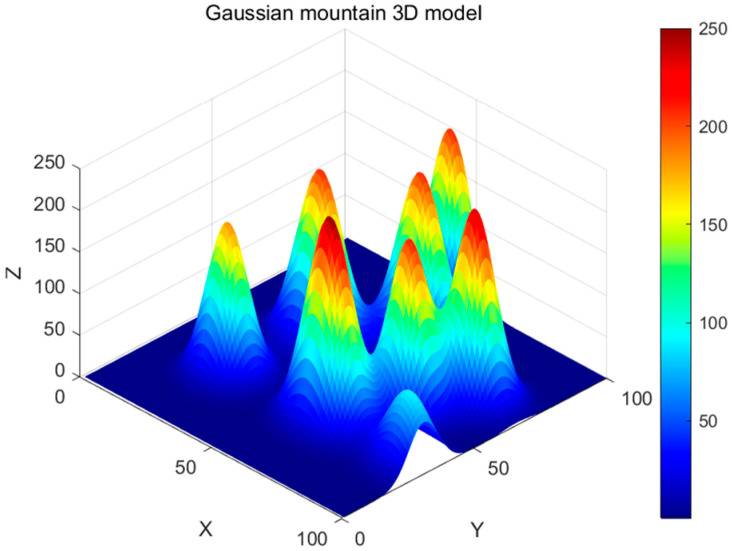
Three-dimensional model of Gaussian mountains.

The specific Gaussian peak modeling is shown in Figure 9.

### 5.2. Flight Path and Smoothing

A Cubic Spline Fitting algorithm is used to generate paths and plot them on a 3D surface. First, global variables are initialized, including the start point, end point, and surface coordinates. Next, the start point, end point, and control points are combined into a sequence to generate a smooth path using cubic spline interpolation, and the original path points are indexed and interpolated. The ‘spline’ function in MATLAB is used to generate the interpolated curves. Finally, ‘surf(X, Y, Z)’ is applied to plot the surface map, ‘shading flat’ removes the grid lines, and ‘colormap’ sets the color mapping. A smooth flight path curve is then drawn.

#### 5.2.1. Cubic Spline Interpolation Path Smoothing Generation Algorithm

In the Improved Lemur Optimization algorithm, each path consists of a starting point, an endpoint, and path points. By finding the optimal positions of the path points, a smooth cubic spline curve is generated by interpolating between adjacent path points.

The flight path for the i-th segment, including n control points, is defined as $f(x_{0})$,$f(x_{1})$, …, $f(x_{n})$ where the domain is defined as $x_{0}<x_{1}<x_{2}<\ldots <x_{n}$. The cubic spline interpolation represents the function between two adjacent path points, and this function, along with its first and second derivatives, is continuous within the interval. The n segmented cubic polynomials are represented as:

(16) $$f_{n}(x)=a_{n}{\left(x-x_{n}\right)}^{3}+b_{n}{\left(x-x_{n}\right)}^{2}+c_{n}(x-x_{n})+d_{n}$$

The n segmented cubic polynomials require solving for 4n unknown parameters: $a_{n},b_{n},c_{n},d_{n}$. Based on the continuity of the derivatives and the interpolation [32], 4n−2 equations can be obtained.

(17) $$\left\{\begin{array}{l} f_{n}(x_{n})\\ f_{n}(x_{n+1})\\ f_{n}^{\prime }(x_{n+1})\\ f_{n}^{\prime \prime }(x_{n+1}) \end{array}\right.$$

**Figure 10:**
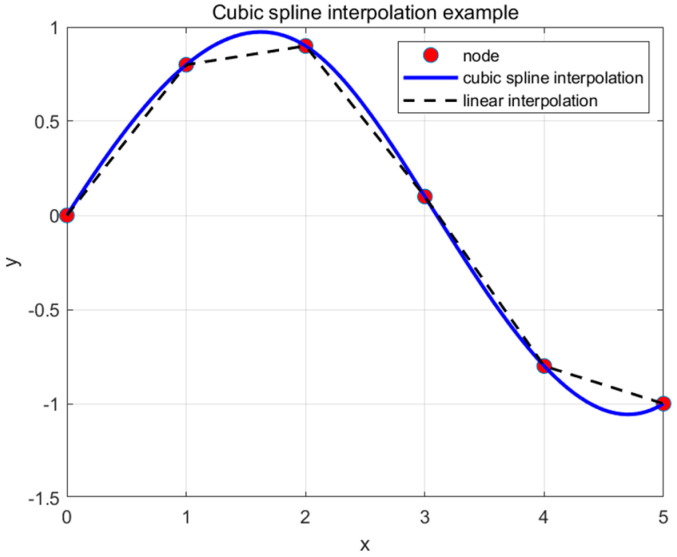
Three times Cubic spline interpolation optimizer effect diagram.

The remaining two conditions are determined by the starting point $x_{0}$ and the target point $x_{n}$. After the UAV completes the path planning, a continuous and smooth cubic spline curve will be generated. The optimization effect of cubic spline interpolation is shown in Figure 10.

**Figure 11:**
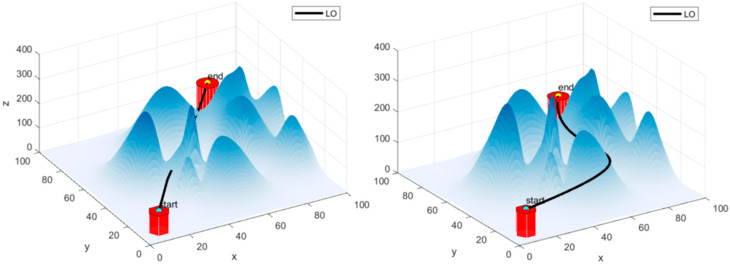
Comparison of generation paths of LO algorithm without Cubic Spline Interpolation Path (**left**) and with Cubic Spline Interpolation Path (**right**) Smoothing algorithm.

Based on Figure 11, the difference between the paths with and without the cubic spline interpolation in the LO algorithm is quite noticeable:

Without cubic spline interpolation (left): The path appears more jagged and less smooth, taking abrupt changes in direction as it navigates the surface. This indicates a direct approach without any smoothing, leading to a less efficient or optimized path.

With cubic spline interpolation (right): The path is much smoother, showing a more gradual and continuous curve. This smoothing effect allows for a more natural and efficient transition along the surface, indicating an improvement in the trajectory planning due to the interpolation.

#### 5.2.2. Restrictive Condition

In order for the UAV to fly in the specified airspace, in each iteration, the position update of the UAV needs to satisfy two conditions:

(1) In order to ensure that the flight path is carried out in the specified airspace, the boundaries need to be constrained and the following constraints need to be satisfied:

(18) $$\left\{\begin{array}{l} 0\leq x_{i}\leq x_{\max}\\ 0\leq y_{i}\leq y_{\max}\\ 0\leq z_{i}\leq z_{\max} \end{array}\right.,i=1,2,\ldots ,n$$

(2) The fitness function [33] out the minimum surrogate value of the flight in the specified airspace that can avoid obstacles, which is derived from the objective function expression with the following expression:

(19) $$fitness=\min(V_{c}+T_{c}+E_{c})$$

#### 5.2.3. Objective Function

The objective function of a UAV flight path is composed of three key elements: total flight distance, obstacle avoidance cost, and the constraint of staying within a specified boundary. These three elements together determine the optimal flight strategy of the UAV. Specifically, the objective function [34,35] considers the following aspects:

(20) $$fitness=V_{c}+T_{c}+E_{c}$$

In Formula (20), $V_{C}$ represents the total flight cost of the UAV; $T_{c}$ represents the cost of the UAV flying around obstacles; $E_{c}$ represents the cost of the UAV flying within the specified boundaries.

The flight cost $V_{c}$ primarily considers the total flight distance of the UAV from the starting point to the endpoint, which is the sum of each arc segment $L_{i}$. If the total flight path consists of *n* segments, the total flight cost can be expressed as:

(21) $$\left\{\begin{array}{l} V_{c}=\overset{n-1}{\underset{i=1}{\sum }}L_{i}\\ L_{i}=\sqrt{{\left(x_{i+1}-x_{i}\right)}^{2}+{\left(y_{i+1}-y_{i}\right)}^{2}+{\left(z_{i+1}-z_{i}\right)}^{2}} \end{array}\right.$$

The terrain cost $T_{c}$ is mainly to ensure that the UAV’s flight path avoids obstacles by controlling the value of $T_{c}$. When the altitude $z_{i}$ is higher than the obstacle height $Z_{2}$($x_{i}$, $y_{i}$) in the terrain, $T_{c}$ = 0; when the altitude $z_{i}$ is lower than the obstacle height $Z_{2}$($x_{i}$, $y_{i}$), $T_{c}$ = ∞. Summing this ensures that the UAV’s flight route avoids obstacles such as peaks. The expression is as follows:

(22) $$\left\{\begin{array}{l} T_{c}=\overset{n}{\underset{i=1}{\sum }}T_{c_{i}}\;T_{c_{0}}=0\\ T_{c_{i}}=\left\{\begin{array}{l} 0\;Z_{i}>Z\left(x_{i},y_{i}\right)\\ \infty \;otherwise\; \end{array}\right. \end{array}\right.$$

The boundary cost $E_{c}$ is to ensure that the UAV remains within the specified airspace, controlled by adjusting the value of $E_{c}$. When the UAV is within the airspace, $E_{c}$ = 0; when it is outside the airspace, $E_{c}$ = ∞. Summing this ensures that the UAV stays within the designated airspace. The expression is as follows:

(23) $$\left\{\begin{array}{l} E_{c}=\overset{n}{\underset{i=1}{\sum }}E_{c_{i}}E_{c_{0}}=0\\ E_{c_{i}}=\left\{\begin{array}{l} \infty \;otherwise\\ 0\;x_{i}\in \left[0,x_{\max}\right]\cap y_{i}\in [0,y_{\max}]\cap z_{i}\in \left[0,z_{\max}\right] \end{array}\right. \end{array}\right.$$

## 6. Analysis of Simulation Results of ILO Algorithm and Other Intelligent Algorithms

In order to verify the effectiveness of the ILO algorithm in the simulation of an UAV mountain 3D path: Set the relevant environmental parameters as shown in Table 3. Where the map uses PSO, GRO, LO, KOA, SWO, and ILO algorithms for the comparison of simulation paths: In order to eliminate the effect of randomness, each algorithm is run independently 100 times on the map to compare its average convergence speed and average adaptation value. The simulation results are shown in Table 4.

As seen in Table 4, the ILO (Improved Lemur Optimization) algorithm performs better in 3D UAV path planning, consistently finding the optimal path quickly and efficiently, regardless of the changing terrain.

**Figure 12:**
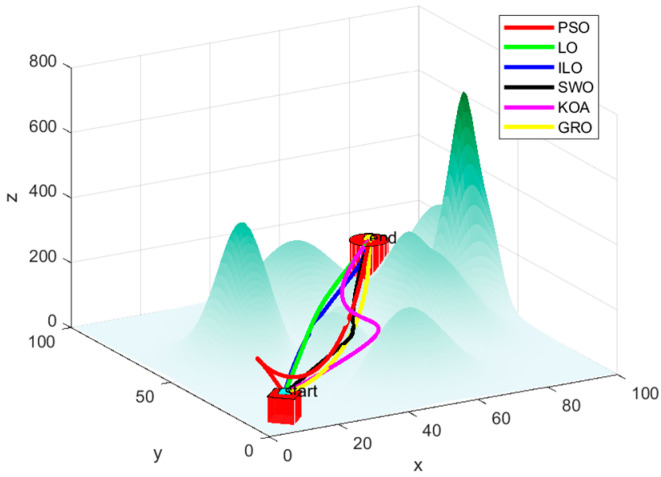
Six algorithms: PSO, LO, ILO, SWO, KOA, and GRO for planning routes.

**Figure 13:**
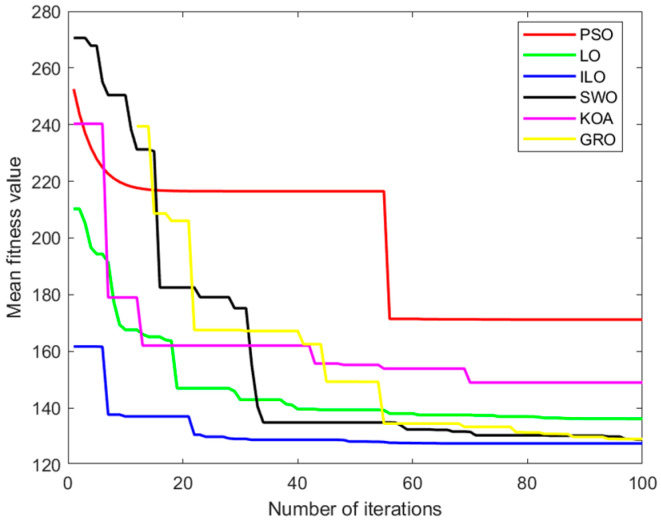
Convergence curves of the six algorithms: PSO, LO, ILO, SWO, KOA, and GRO.

**Figure 14:**
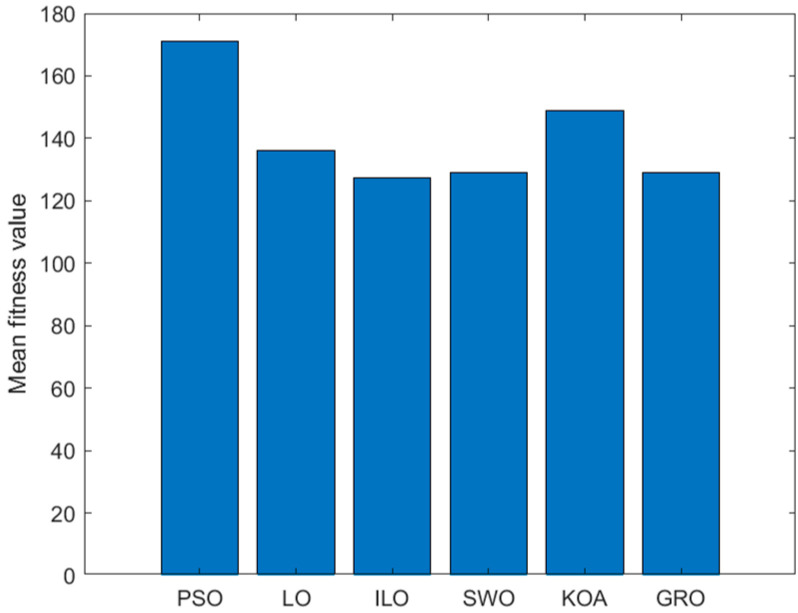
Histogram of the path lengths of the six algorithms: PSO, LO, ILO, SWO, KOA, and GRO.

In Figure 12, Figure 13 and Figure 14, the PSO algorithm’s path shows some smoothness but appears somewhat volatile, especially near the endpoint, where it may become unstable. The path tends to get stuck in local optima in certain regions, highlighting the local difficulties PSO might encounter in complex search spaces. The LO algorithm’s path is more winding, with noticeable turning points in some areas. Although it finds a reasonably good endpoint, its performance is slightly worse than the improved ILO, showing some local oscillations. In contrast, ILO’s path is relatively smooth, directly connecting the start and endpoint, which indicates that the ILO algorithm is more robust during the search process and better at avoiding local optima. ILO demonstrates more efficient convergence and superior global search capability when solving the path planning problem.

The SWO algorithm performs moderately, with no significant oscillations and a certain degree of smoothness, but its overall performance lags behind ILO. KOA also shows moderate performance, similar to SWO. Although it can find the target endpoint, its path is not as smooth as ILO or PSO. The GRO shows relatively good performance, but compared to ILO, its path experiences minor fluctuations and oscillations in the middle, indicating that it might become stuck in local optima in certain regions, causing the path to deviate slightly.

Compared to other algorithms, ILO converges faster, with the fitness value reaching a lower level. Around the 30th iteration, the fitness value stabilizes, indicating that the algorithm finds a good solution in a relatively short time.

## 7. Concluding Remarks

This paper introduces an enhanced Improved Lemur Optimization algorithm (ILO), which demonstrates exceptional performance in UAV path planning, primarily due to its multi-tiered optimization framework. In the initial phases, the ILO algorithm modifies the jump rate to enable broader exploration of the search space, progressively reducing this rate in later stages to facilitate more precise local optimization. Furthermore, the algorithm incorporates elements from the Spider Wasp Optimization Algorithm, utilizing three distinct behaviors to dynamically decrease population size, thereby accelerating convergence. The integration of the Levy flight mechanism allows for significant leaps in the search process, substantially enhancing global exploration capabilities. An adaptive nonlinear decreasing model is employed to further refine the jump rate and reduce crossover probability, thereby improving the efficiency of local searches. When combined with simulated annealing, the algorithm effectively circumvents entrapment in local optima, while the inclusion of adaptive learning factors bolsters its iterative and search capabilities. Ultimately, the ILO algorithm achieves rapid and precise path planning optimization through a synergistic blend of these multiple strategies. While the ILO approach results in superior UAV route planning quality, it does increase computational complexity. Future research could focus on enhancing the algorithm’s performance and efficiency while preserving the streamlined nature of its mechanisms. The ILO method holds significant potential for future applications, including collaborative UAV path planning and dynamic collision avoidance.

## Figures and Tables

**Table 1:** Summary of the CEC’17 test functions [27].

	No.	Functions	Fi* = Fi(x*)
Unimodal Functions	1	Shifted and Rotated Bent Cigar Function	100
	2	Shifted and Rotated Sum of Different Power Function	200
	3	Shifted and Rotated Zakharov Function	300
Simple Multimodal Functions	4	Shifted and Rotated Rosenbrock’s Function	400
	5	Shifted and Rotated Rastrigin’s Function	500
	6	Shifted and Rotated Expanded Scaffer’s F6 Function	600
	7	Shifted and Rotated Lunacek Bi_Rastrigin Function	700
	8	Shifted and Rotated Non-Continuous Rastrigin’s Function	800
	9	Shifted and Rotated Levy Function	900
	10	Shifted and Rotated Schwefel’s Function	1000
Hybrid Functions	11	Hybrid Function 1 (*N* = 3)	1100
	12	Hybrid Function 2 (*N* = 3)	1200
	13	Hybrid Function 3 (*N* = 3)	1300
	14	Hybrid Function 4 (*N* = 4)	1400
	15	Hybrid Function 5 (*N* = 4)	1500
	16	Hybrid Function 6 (*N* = 4)	1600
	17	Hybrid Function 6 (*N* = 5)	1700
	18	Hybrid Function 6 (*N* = 5)	1800
	19	Hybrid Function 6 (*N* = 5)	1900
	20	Hybrid Function 6 (*N* = 6)	2000
Composition Functions	21	Composition Function 1 (*N* = 3)	2100
	22	Composition Function 2 (*N* = 3)	2200
	23	Composition Function 3 (*N* = 4)	2300
	24	Composition Function 4 (*N* = 4)	2400
	25	Composition Function 5 (*N* = 5)	2500
	26	Composition Function 6 (*N* = 5)	2600
	27	Composition Function 7 (*N* = 6)	2700
	28	Composition Function 8 (*N* = 6)	2800
	29	Composition Function 9 (*N* = 3)	2900
	30	Composition Function 10 (*N* = 3)	3000
Search Range: ${\left[-100,100\right]}^{D}$			

**Table 2:** Algorithm parameters.

Algorithm	Population	Size Number of Iterations	Parameters
GRO	30	500	$\mathrm{Iter}=1,\;\sigma_{0}$ = 2
PSO	30	500	$w=0.8,\;c_{1}$$=1.5,\;c_{2}$ = 1.5
SWO	30	500	$\mathrm{TR}=0.3,\;\mathrm{Cr}=0.2,\;N_{min}$ = 20, t = 0
KOA	30	500	$T_{0}$ $=3,\;M_{0}$ = 0.1, λ= 15
LO	30	500	Iter = 0, jumping rate min = 0.1 Jumping rate max = 0.5
ILO	30	500	Initial jumping rate = 0.5 Jumping rate min = 0.1 Jumping rate max = 0.5 $N_{min}$ $=20,\;\mathrm{TR}=0.3,\;\mathrm{Cr}=0.2,\;T_{0}$ =100, α = 0.95

**Table 3:** Environmental parameters.

	Parameters	Notation	Parameter Value
Map	Execution space (math.)		100 × 100 × 250
	Starting point	Start	[10,10,10]
	Target point	Goal	[80,90,80]
	Number of peaks	N	8
	Population size	SearchAgents_no	30
	Number of iterations	Iter	100

**Table 4:** Comparison of average fitness value and convergence speed.

Scales	Algorithms	Average Number of Convergence Iterations	Mean Fitness Value	Percentage of ILO Adaptation Values/%	Percentage of ILO Converged Iterations/%
Map	PSO	56	171.2	98.3	74.4
	LO	86	136.2	64.0	93.5
	ILO	55	127.4	100	100
	SWO	97	129.1	56.7	98.7
	KOA	70	149.0	78.6	85.5
	GRO	94	129.1	58.5	98.7

## Data Availability

The raw data supporting the conclusions of this article will be made available by the authors on request.

## Appendix A

**Table A1:** Comparison of 6 algorithms of 50 dimension.

		Dim = 50					
		GRO	LO	PSO	SWO	KOA	ILO
F1	Max	6.95E+09	3.08E+08	1.61E+10	9.78E+08	8.25E+07	6.32E+04
	Mean	3.37E+09	2.121E+08	4.41E+09	4.70E+08	2.21E+07	1.65E+04
	Min	1.52E+09	1.02E+08	2.86E+07	2.28E+08	2.20E+06	1.92E+03
	*p*-value	3.02E−11	3.02E−11	3.02E−11	3.02E−11	3.02E−11	1
	Std	1.36E+09	6.25E+07	3.80E+09	1.95E+08	1.82E+07	1.46E+04
F2	Max	3.58E+54	5.45E+64	4.19E+55	2.68E+48	1.39E+45	3.22E+40
	Mean	1.40E+53	2.59E+63	1.54E+54	1.02E+47	9.03E+43	1.14E+39
	Min	4.97E+44	8.31E+48	4.29E+37	1.95E+39	1.62E+32	3.08E+30
	*p*-value	3.02E−11	3.02E−11	2.61E−10	4.50E−11	3.32E−06	1
	Std	6.56E+53	1.05E+64	7.65E+54	4.89E+47	3.18E+44	5.88E+39
F3	Max	2.10E+05	4.53E+05	4.63E+05	1.78E+05	2.86E+05	1.60E+05
	Mean	1.64E+05	3.21E+05	2.19E+05	1.17E+05	2.02E+05	1.03E+05
	Min	1.15E+05	2.40E+05	1.23E+05	8.02E+04	1.29E+05	5.18E+04
	*p*-value	3.96E−08	3.02E−11	8.10E−10	7.98E−02	8.99E−11	1
	Std	2.74E+04	5.31E+04	7.64E+04	2.14E+04	3.65E+04	3.32E+04
F4	Max	1.66E+03	8.44E+02	2.80E+04	8.47E+02	7.71E+02	6.60E+02
	Mean	1.05E+03	7.20E+02	9.16E+02	7.46E+02	6.70E+02	5.74E+02
	Min	8.41E+02	5.98E+02	5.88E+02	6.96E+02	5.52E+02	4.55E+02
	*p*-value	3.02E−11	1.46E−10	3.82E−10	3.02E−11	2.20E−07	1
	Std	1.73E+02	5.70E+01	4.74E+02	4.13E+01	6.30E+01	4.60E+01
F5	Max	8.45E+02	9.77E+02	9.27E+02	8.74E+02	9.91E+02	6.52E+02
	Mean	7.94E+02	8.97E+02	7.96E+02	7.52E+02	9.34E+02	5.91E+02
	Min	7.37E+02	7.98E+02	6.65E+02	6.90E+02	8.66E+02	5.57E+02
	*p*-value	3.02E−11	3.02E−11	3.02E−11	3.02E−11	3.02E−11	1
	Std	2.73E+01	4.76E+01	6.40E+01	4.57E+01	2.98E+01	2.72E+01
F6	Max	6.29E+02	6.16E+02	6.53E+02	6.21E+02	6.26E+02	6.06E+02
	Mean	6.20E+02	6.09E+02	6.35E+02	6.16E+02	6.12E+02	6.04E+02
	Min	6.14E+02	6.05E+02	6.14E+02	6.11E+02	6.06E+02	6.01E+02
	*p*-value	3.02E−11	6.07E−11	3.02E−11	3.02E−11	4.08E−11	1
	Std	4.11E+00	2.58E+00	8.29E+00	2.78E+00	4.77E+00	1.17E+00
F7	Max	1.23E+03	1.34E+03	1.51E+03	1.29E+03	1.41E+03	1.01E+03
	Mean	1.10E+03	1.22E+03	1.19E+03	1.14E+03	1.27E+03	8.96E+02
	Min	9.52E+02	1.13E+03	1.00E+03	9.73E+02	1.16E+03	8.38E+02
	*p*-value	5.49E−11	3.02E−11	3.34E−11	3.69E−11	3.02E−11	1
	Std	6.11E+01	5.23E+01	1.10E+02	7.63E+01	4.85E+01	4.53E+01
F8	Max	1.21E+03	1.28E+03	1.18E+03	1.10E+03	1.32E+03	9.66E+02
	Mean	1.11E+03	1.18E+03	1.07E+03	1.04E+03	1.24E+03	8.98E+02
	Min	1.03E+03	1.08E+03	9.44E+02	9.72E+02	1.19E+03	8.50E+02
	*p*-value	3.02E−11	3.02E−11	3.34E−11	3.02E−11	3.02E−11	1
	Std	3.79E+01	4.82E+01	5.94E+01	4.07E+01	3.00E+01	2.63E+01
F9	Max	6.93E+03	1.47E+04	3.58E+04	1.93E+04	1.39E+04	3.73E+03
	Mean	4.75E+03	6.71E+03	1.87E+04	9.60E+03	6.86E+03	1.99E+03
	Min	2.66E+03	3.12E+03	4.85E+03	3.64E+03	2.80E+03	1.15E+03
	*p*-value	1.33E−10	3.69E−11	3.02E−11	3.34E−11	8.99E−11	1
	Std	1.23E+03	2.56E+03	7.37E+03	3.90E+03	2.73E+03	6.77E+02
F10	Max	1.23E+04	1.54E+04	9.65E+03	1.28E+04	1.48E+04	1.07E+04
	Mean	1.09E+04	1.41E+04	7.76E+03	9.24E+03	1.41E+04	8.64E+03
	Min	9.20E+03	1.24E+04	5.64E+03	6.85E+03	1.31E+04	7.02E+03
	*p*-value	1.17E−09	3.02E−11	3.18E−03	7.48E−02	3.02E−11	1
	Std	8.34E+02	7.24E+02	9.76E+02	1.36E+03	4.32E+02	9.89E+02
F11	Max	4.04E+03	1.13E+04	3.20E+03	2.27E+03	1.93E+03	1.58E+03
	Mean	3.09E+03	5.24E+03	1.71E+03	1.78E+03	1.67E+03	1.39E+03
	Min	2.03E+03	2.08E+03	1.38E+03	1.48E+03	1.44E+03	1.26E+03
	*p*-value	3.02E−11	3.02E−11	6.12E−10	9.92E−11	1.33E−10	1
	Std	5.05E+02	2.34E+03	3.11E+02	2.27E+02	1.19E+02	7.39E+01
F12	Max	2.82E+08	1.997E+08	4.205E+09	7.78E+07	6.86E+07	9.47E+06
	Mean	1.32E+08	8.32E+07	1.23E+09	2.66E+07	1.17E+07	3.88E+06
	Min	3.67E+07	2.09E+07	9.76E+06	5.26E+06	2.74E+06	1.57E+06
	*p*-value	3.02E−11	3.02E−11	3.02E−11	6.07E−11	6.53E−08	1
	Std	5.57E+07	4.50E+07	1.18E+09	1.37E+07	1.18E+07	1.91E+06
F13	Max	3.81E+06	2.99E+04	6.11E+09	5.58E+04	2.81E+04	2.00E+04
	Mean	1.14E+06	1.10E+04	4.56E+08	2.31E+04	7.82E+03	6.02E+03
	Min	3.44E+04	2.50E+03	7.72E+03	8.00E+03	3.40E+03	1.67E+03
	*p*-value	3.02E−11	1.95E−03	1.46E−10	1.17E−09	1.27E−02	1
	Std	1.07E+06	7.89E+03	1.17E+09	1.09E+04	4.96E+03	4.92E+03
F14	Max	8.46E+05	5.26E+06	5.60E+06	1.62E+06	1.71E+05	2.24E+05
	Mean	3.11E+05	1.36E+06	1.02E+06	2.55E+05	8.18E+04	5.51E+04
	Min	7.45E+04	1.26E+05	1.33E+05	1.80E+04	2.26E+04	2.83E+03
	*p*-value	1.17E−09	4.50E−11	4.50E−11	2.88E−06	1.08E−02	1
	Std	1.79E+05	1.24E+06	1.07E+06	3.00E+05	4.00E+04	5.22E+04
F15	Max	7.25E+05	2.23E+04	1.12E+05	2.19E+04	1.07E+05	1.93E+04
	Mean	9.23E+04	1.13E+04	2.26E+04	9.91E+03	1.27E+04	6.08E+03
	Min	8.98E+03	1.84E+03	2.46E+03	2.83E+03	2.31E+03	2.02E+03
	*p*-value	3.82E−10	9.47E−03	1.61E−06	1.30E−03	3.85E−03	1
	Std	1.55E+05	6.73E+03	2.19E+04	5.66E+03	1.85E+04	4.24E+03
F16	Max	4.41E+03	5.28E+03	4.70E+03	4.29E+03	5.46E+03	4.22E+03
	Mean	3.36E+03	4.45E+03	3.44E+03	3.29E+03	4.86E+03	3.24E+03
	Min	2.43E+03	3.31E+03	2.60E+03	2.40E+03	4.03E+03	2.10E+03
	*p*-value	4.29E−01	8.10E−10	2.40E−01	8.42E−01	3.34E−11	1
	Std	4.18E+02	4.63E+02	4.74E+02	4.55E+02	3.43E+02	4.83E+02
F17	Max	3.55E+03	4.14E+03	3.72E+03	3.46E+03	4.13E+03	3.86E+03
	Mean	2.94E+03	3.63E+03	3.26E+03	2.98E+03	3.72E+03	3.14E+03
	Min	2.28E+03	3.27E+03	2.59E+03	2.53E+03	2.99E+03	2.38E+03
	*p*-value	1.56E−02	1.16E−07	1.71E−01	1.12E−02	1.85E−08	1
	Std	3.03E+02	2.76E+02	3.21E+02	2.31E+02	2.67E+02	3.04E+02
F18	Max	1.02E+07	7.17E+07	1.88E+07	8.13E+06	5.94E+06	2.20E+06
	Mean	3.96E+06	1.45E+07	5.20E+06	1.44E+06	1.51E+06	6.83E+05
	Min	7.45E+05	1.77E+06	4.23E+05	1.61E+05	3.20E+05	5.64E+04
	*p*-value	1.07E−09	4.08E−11	5.46E−09	2.15E−02	2.68E−04	1
	Std	2.67E+06	1.39E+07	4.18E+06	1.57E+06	1.25E+06	5.42E+05
F19	Max	8.01E+05	4.43E+04	1.89E+06	4.01E+04	4.11E+04	4.03E+04
	Mean	9.15E+04	1.81E+04	1.22E+05	1.76E+04	2.04E+04	1.73E+04
	Min	1.45E+04	2.00E+03	2.09E+03	5.40E+03	2.79E+03	2.05E+03
	*p*-value	2.00E−06	9.71E−01	1.26E−01	9.23E−01	2.28E−01	1
	Std	1.54E+05	1.28E+04	3.51E+05	1.02E+04	1.02E+04	9.92E+03
F20	Max	3.41E+03	4.11E+03	3.65E+03	3.52E+03	4.33E+03	3.84E+03
	Mean	2.97E+03	3.63E+03	3.10E+03	2.99E+03	4.01E+03	3.19E+03
	Min	2.51E+03	2.98E+03	2.44E+03	2.41E+03	3.42E+03	2.69E+03
	*p*-value	6.97E−03	5.09E−06	3.40E−01	1.44E−02	1.09E−10	1
	Std	2.46E+02	2.89E+02	3.06E+02	2.77E+02	1.95E+02	3.17E+02
F21	Max	2.64E+03	2.77E+03	2.76E+03	2.63E+03	2.76E+03	2.46E+03
	Mean	2.57E+03	2.69E+03	2.60E+03	2.55E+03	2.71E+03	2.40E+03
	Min	2.51E+03	2.61E+03	2.51E+03	2.48E+03	2.67E+03	2.36E+03
	*p*-value	3.02E−11	3.02E−11	3.02E−11	3.02E−11	3.02E−11	1
	Std	3.23E+01	3.61E+01	6.04E+01	4.23E+01	2.84E+01	2.46E+01
F22	Max	1.37E+04	1.66E+04	1.18E+04	1.38E+04	1.70E+04	1.16E+04
	Mean	1.12E+04	1.54E+04	9.64E+03	1.01E+04	1.56E+04	9.53E+03
	Min	2.89E+03	1.40E+04	2.77E+03	2.68E+03	8.85E+03	8.13E+03
	*p*-value	1.25E−05	3.02E−11	2.06E−01	4.23E−03	2.37E−10	1
	Std	3.22E+03	6.46E+02	1.66E+03	2.64E+03	1.35E+03	9.73E+02
F23	Max	3.10E+03	3.19E+03	3.72E+03	3.23E+03	3.25E+03	2.92E+03
	Mean	3.03E+03	3.10E+03	3.37E+03	3.05E+03	3.17E+03	2.85E+03
	Min	2.97E+03	3.01E+03	3.06E+03	2.96E+03	3.10E+03	2.77E+03
	*p*-value	3.02E−11	3.02E−11	3.02E−11	3.02E−11	3.02E−11	1
	Std	3.09E+01	4.56E+01	1.76E+02	5.68E+01	3.10E+01	3.28E+01
F24	Max	3.33E+03	3.36E+03	3.85E+03	3.33E+03	3.44E+03	3.15E+03
	Mean	3.21E+03	3.29E+03	3.54E+03	3.22E+03	3.33E+03	3.01E+03
	Min	3.14E+03	3.21E+03	3.31E+03	3.11E+03	3.28E+03	2.95E+03
	*p*-value	4.08E−11	3.02E−11	3.02E−11	4.50E−11	3.02E−11	1
	Std	3.90E+01	3.58E+01	1.38E+02	5.42E+01	4.04E+01	4.09E+01
F25	Max	4.48E+03	3.41E+03	3.41E+03	3.39E+03	3.26E+03	3.12E+03
	Mean	3.50E+03	3.22E+03	3.17E+03	3.27E+03	3.16E+03	3.07E+03
	Min	3.25E+03	3.12E+03	3.03E+03	3.12E+03	3.04E+03	3.03E+03
	*p*-value	3.02E−11	3.02E−11	1.01E−08	3.02E−11	2.03E−09	1
	Std	2.23E+02	6.41E+01	8.28E+01	7.65E+01	4.66E+01	2.29E+01
F26	Max	8.07E+03	8.50E+03	9.30E+03	8.04E+03	9.81E+03	5.99E+03
	Mean	6.88E+03	7.40E+03	7.03E+03	7.33E+03	8.29E+03	5.09E+03
	Min	5.74E+03	6.31E+03	**3.57E+03**	6.27E+03	7.52E+03	4.55E+03
	*p*-value	3.69E−11	3.02E−11	1.43E−05	3.02E−11	3.02E−11	1
	Std	4.92E+02	4.67E+02	1.65E+03	5.00E+02	4.63E+02	3.54E+02
F27	Max	3.99E+03	3.79E+03	4.10E+03	3.99E+03	3.73E+03	3.57E+03
	Mean	3.81E+03	3.49E+03	3.70E+03	3.71E+03	3.50E+03	3.40E+03
	Min	3.63E+03	3.37E+03	3.39E+03	3.50E+03	3.33E+03	3.28E+03
	*p*-value	3.02E−11	5.09E−06	1.20E−08	4.98E−11	2.00E−05	1
	Std	8.95E+01	7.81E+01	2.00E+02	1.23E+02	9.32E+01	6.33E+01
F28	Max	4.32E+03	4.52E+03	7.06E+03	4.75E+03	3.72E+03	3.45E+03
	Mean	3.99E+03	3.77E+03	4.03E+03	3.85E+03	3.52E+03	3.36E+03
	Min	3.65E+03	3.45E+03	3.38E+03	3.53E+03	3.38E+03	3.29E+03
	*p*-value	3.02E−11	3.02E−11	1.33E−10	3.02E−11	1.46E−10	1
	Std	1.62E+02	2.88E+02	8.93E+02	2.48E+02	7.53E+01	3.53E+01
F29	Max	5.35E+03	5.67E+03	5.90E+03	4.99E+03	5.68E+03	5.54E+03
	Mean	4.58E+03	4.77E+03	4.69E+03	4.53E+03	5.03E+03	4.44E+03
	Min	4.03E+03	4.06E+03	3.86E+03	3.99E+03	4.04E+03	3.50E+03
	*p*-value	1.30E−01	1.60E−03	2.71E−02	3.18E−01	8.29E−06	1
	Std	3.32E+02	3.70E+02	4.40E+02	2.82E+02	4.45E+02	4.04E+02
F30	Max	6.07E+07	2.96E+06	2.16E+07	1.18E+07	9.10E+06	2.36E+06
	Mean	2.39E+07	1.48E+06	4.45E+06	6.84E+06	4.21E+06	1.19E+06
	Min	8.69E+06	8.82E+05	9.03E+05	3.15E+06	1.49E+06	7.53E+05
	*p*-value	3.02E−11	1.11E−03	2.38E−07	3.02E−11	2.15E−10	1
	Std	1.39E+07	4.46E+05	4.80E+06	2.06E+06	2.15E+06	4.10E+05

## Appendix B

**Table A2:** Comparison of 6 algorithms of 100 dimension.

		Dim = 100					
		GRO	LO	PSO	SWO	KOA	ILO
F1	Max	7.10E+10	2.62E+10	5.54E+10	2.81E+10	8.21E+09	3.26E+08
	Mean	5.33E+10	1.72E+10	1.89E+10	1.92E+10	4.12E+09	9.10E+07
	Min	3.67E+10	1.25E+10	5.88E+09	1.14E+10	1.83E+09	2.40E+07
	*p*-value	3.02E−11	3.02E−11	3.02E−11	3.02E−11	3.02E−11	1
	Std	8.28E+09	3.06E+09	1.13E+10	4.59E+09	1.48E+09	6.82E+07
F2	Max	4.97E+134	3.31E+154	1.97E+151	4.75E+128	1.47E+126	4.76E+116
	Mean	2.14E+133	1.10E+153	6.57E+149	3.34E+127	4.89E+124	1.59E+115
	Min	2.48E+118	2.57E+130	1.06E+105	2.70E+112	3.19E+106	4.88E+93
	*p*-value	3.02E−11	3.02E−11	8.10E−10	3.69E−11	3.50E−09	1
	Std	9.21E+133	Infinity	3.60E+150	1.00E+128	2.67E+125	8.69E+115
F3	Max	6.03E+05	9.52E+05	8.15E+05	3.86E+05	7.48E+05	4.24E+05
	Mean	4.49E+05	8.25E+05	6.29E+05	3.25E+05	5.68E+05	3.05E+05
	Min	3.05E+05	6.17E+05	3.98E+05	2.57E+05	4.16E+05	2.32E+05
	*p*-value	2.67E−09	3.02E−11	4.98E−11	1.22E−02	3.69E−11	1
	Std	6.84E+04	7.93E+04	9.60E+04	2.79E+04	8.58E+04	5.85E+04
F4	Max	7.23E+03	4.51E+03	7.58E+03	3.81E+03	2.15E+03	1.06E+03
	Mean	5.80E+03	2.76E+03	2.71E+03	2.73E+03	1.57E+03	9.27E+02
	Min	4.61E+03	1.84E+03	1.29E+03	1.97E+03	1.28E+03	8.02E+02
	*p*-value	3.02E−11	3.02E−11	3.02E−11	3.02E−11	3.02E−11	1
	Std	7.03E+02	5.49E+02	1.46E+03	4.68E+02	1.86E+02	6.70E+01
F5	Max	1.59E+03	1.71E+03	1.72E+03	1.51E+03	1.82E+03	9.17E+02
	Mean	1.40E+03	1.59E+03	1.41E+03	1.33E+03	1.66E+03	8.37E+02
	Min	1.26E+03	1.46E+03	1.17E+03	1.14E+03	1.56E+03	7.26E+02
	*p*-value	3.02E−11	3.02E−11	3.02E−11	3.02E−11	3.02E−11	1
	Std	7.30E+01	7.41E+01	1.24E+02	9.09E+01	6.67E+01	5.05E+01
F6	Max	6.65E+02	6.47E+02	6.81E+02	6.55E+02	6.63E+02	6.29E+02
	Mean	6.47E+02	6.37E+02	6.62E+02	6.43E+02	6.44E+02	6.20E+02
	Min	6.36E+02	6.26E+02	6.36E+02	6.33E+02	6.31E+02	6.11E+02
	*p*-value	3.02E−11	4.08E−11	3.02E−11	3.02E−11	3.02E−11	1
	Std	5.64E+00	4.96E+00	9.89E+00	6.26E+00	7.43E+00	4.39E+00
F7	Max	2.58E+03	2.99E+03	2.61E+03	2.70E+03	2.87E+03	1.93E+03
	Mean	2.24E+03	2.59E+03	2.24E+03	2.39E+03	2.49E+03	1.54E+03
	Min	1.94E+03	2.24E+03	1.99E+03	2.01E+03	2.19E+03	1.32E+03
	*p*-value	3.02E−11	3.02E−11	3.02E−11	3.02E−11	3.02E−11	1
	Std	1.45E+02	1.73E+02	1.50E+02	1.73E+02	1.70E+02	1.30E+02
F8	Max	1.88E+03	2.07E+03	2.11E+03	1.89E+03	2.11E+03	1.29E+03
	Mean	1.74E+03	1.88E+03	1.77E+03	1.67E+03	1.95E+03	1.16E+03
	Min	1.62E+03	1.69E+03	1.58E+03	1.46E+03	1.83E+03	1.05E+03
	*p*-value	3.02E−11	3.02E−11	3.02E−11	3.02E−11	3.02E−11	1
	Std	7.11E+01	6.67E+01	1.40E+02	1.15E+02	6.24E+01	5.94E+01
F9	Max	4.28E+04	7.08E+04	9.86E+04	7.32E+04	6.66E+04	2.10E+04
	Mean	3.12E+04	4.60E+04	6.97E+04	4.29E+04	4.13E+04	1.14E+04
	Min	1.92E+04	3.44E+04	3.67E+04	2.76E+04	2.56E+04	6.64E+03
	*p*-value	4.08E−11	3.02E−11	3.02E−11	3.02E−11	3.02E−11	1
	Std	6.51E+03	7.79E+03	1.55E+04	1.15E+04	9.65E+03	3.57E+03
F10	Max	2.86E+04	3.34E+04	2.70E+04	3.00E+04	3.25E+04	2.39E+04
	Mean	2.60E+04	3.15E+04	2.05E+04	2.40E+04	3.14E+04	1.79E+04
	Min	2.31E+04	2.94E+04	1.71E+04	2.04E+04	3.01E+04	1.55E+04
	*p*-value	3.69E−11	3.02E−11	9.51E−06	2.15E−10	3.02E−11	1
	Std	1.49E+03	9.59E+02	2.26E+03	2.62E+03	5.09E+02	1.86E+03
F11	Max	1.44E+05	3.33E+05	1.20E+05	8.13E+04	1.30E+05	8.25E+04
	Mean	1.03E+05	2.22E+05	6.96E+04	5.74E+04	9.35E+04	2.36E+04
	Min	6.48E+04	1.38E+05	3.29E+04	2.10E+04	6.16E+04	1.16E+04
	*p*-value	4.98E−11	3.02E−11	5.57E−10	2.44E−09	8.15E−11	1
	Std	1.92E+04	5.04E+04	2.04E+04	1.21E+04	1.98E+04	1.43E+04
F12	Max	7.23E+09	3.39E+09	2.44E+10	2.40E+09	7.37E+08	1.37E+08
	Mean	4.77E+09	2.12E+09	8.48E+09	1.07E+09	3.81E+08	5.08E+07
	Min	1.89E+09	9.26E+08	1.33E+09	6.84E+08	1.81E+08	1.42E+07
	*p*-value	3.02E−11	3.02E−11	3.02E−11	3.02E−11	3.02E−11	1
	Std	1.48E+09	6.10E+08	6.55E+09	3.55E+08	1.31E+08	2.63E+07
F13	Max	3.01E+08	1.69E+05	6.27E+09	3.49E+06	1.76E+06	2.97E+04
	Mean	7.36E+07	8.99E+04	1.10E+09	1.08E+06	9.15E+04	8.24E+03
	Min	1.76E+07	4.53E+04	9.77E+04	2.70E+05	1.98E+04	3.08E+03
	*p*-value	3.02E−11	3.02E−11	3.02E−11	3.02E−11	9.92E−11	1
	Std	6.13E+07	3.26E+04	1.54E+09	7.45E+05	3.15E+05	6.30E+03
F14	Max	1.40E+07	6.73E+07	2.55E+07	4.66E+06	8.31E+06	3.30E+06
	Mean	6.79E+06	2.51E+07	6.51E+06	2.43E+06	3.35E+06	1.32E+06
	Min	1.65E+06	8.63E+06	1.03E+06	8.69E+05	1.41E+06	2.39E+05
	*p*-value	1.33E−10	3.02E−11	5.46E−09	1.64E−05	2.19E−08	1
	Std	3.21E+06	1.27E+07	5.27E+06	9.92E+05	1.66E+06	7.44E+05
F15	Max	5.52E+06	4.41E+05	1.38E+09	5.11E+04	1.72E+04	2.31E+04
	Mean	1.82E+06	2.40E+04	1.85E+08	2.14E+04	6.21E+03	5.83E+03
	Min	1.70E+05	4.03E+03	9.86E+03	9.28E+03	3.72E+03	2.26E+03
	*p*-value	3.02E−11	1.86E−03	7.39E−11	1.55E−09	3.03E−02	1
	Std	1.42E+06	7.92E+04	4.25E+08	9.76E+03	2.92E+03	4.86E+03
F16	Max	9.34E+03	1.20E+04	7.22E+03	7.98E+03	1.11E+04	7.57E+03
	Mean	7.93E+03	1.04E+04	6.27E+03	7.00E+03	1.00E+04	5.58E+03
	Min	6.59E+03	8.89E+03	5.07E+03	5.30E+03	8.06E+03	4.34E+03
	*p*-value	7.39E−11	3.02E−11	2.68E−04	4.69E−08	3.02E−11	1
	Std	6.86E+02	7.24E+02	6.05E+02	7.11E+02	7.15E+02	7.30E+02
F17	Max	6.77E+03	8.23E+03	2.62E+04	6.55E+03	8.01E+03	6.54E+03
	Mean	5.49E+03	7.39E+03	6.73E+03	5.39E+03	7.21E+03	5.19E+03
	Min	4.74E+03	6.35E+03	4.30E+03	4.47E+03	6.18E+03	3.63E+03
	*p*-value	1.08E−02	3.34E−11	3.37E−05	1.30E−01	4.08E−11	1
	Std	4.06E+02	4.66E+02	3.85E+03	4.73E+02	4.48E+02	5.88E+02
F18	Max	1.95E+07	9.43E+07	2.11E+07	9.63E+06	1.90E+07	4.54E+06
	Mean	9.00E+06	4.41E+07	8.02E+06	3.50E+06	7.01E+06	2.28E+06
	Min	1.96E+06	1.41E+07	2.75E+06	1.32E+06	1.79E+06	2.63E+05
	*p*-value	1.96E−10	3.02E−11	1.33E−10	9.88E−03	1.43E−08	1
	Std	3.73E+06	2.17E+07	4.07E+06	1.88E+06	3.82E+06	1.10E+06
F19	Max	1.60E+07	1.89E+04	5.20E+09	2.37E+05	1.53E+04	1.29E+04
	Mean	2.89E+06	1.02E+04	3.28E+08	6.99E+04	5.79E+03	4.96E+03
	Min	4.88E+05	3.07E+03	1.43E+04	7.45E+03	2.69E+03	2.17E+03
	*p*-value	3.02E−11	1.02E−05	3.02E−11	4.50E−11	7.48E−02	1
	Std	3.05E+06	4.95E+03	9.52E+08	5.77E+04	2.97E+03	3.08E+03
F20	Max	6.39E+03	8.28E+03	6.37E+03	6.94E+03	8.20E+03	6.68E+03
	Mean	5.70E+03	7.30E+03	5.45E+03	5.32E+03	7.72E+03	5.62E+03
	Min	4.35E+03	6.11E+03	4.29E+03	3.87E+03	6.85E+03	3.40E+03
	*p*-value	6.31E−01	2.37E−10	2.12E−01	3.64E−02	3.02E−11	1
	Std	4.44E+02	5.54E+02	6.02E+02	6.16E+02	3.07E+02	7.02E+02
F21	Max	3.36E+03	3.71E+03	4.11E+03	3.35E+03	3.56E+03	2.82E+03
	Mean	3.19E+03	3.45E+03	3.50E+03	3.15E+03	3.43E+03	2.67E+03
	Min	3.07E+03	3.31E+03	3.21E+03	3.02E+03	3.32E+03	2.57E+03
	*p*-value	3.02E−11	3.02E−11	3.02E−11	3.02E−11	3.02E−11	1
	Std	6.64E+01	8.73E+01	1.94E+02	7.59E+01	6.36E+01	6.41E+01
F22	Max	3.04E+04	3.57E+04	3.45E+04	3.22E+04	3.46E+04	2.52E+04
	Mean	2.88E+04	3.36E+04	2.49E+04	2.57E+04	3.39E+04	2.05E+04
	Min	2.73E+04	3.15E+04	1.98E+04	2.10E+04	3.04E+04	1.64E+04
	*p*-value	3.02E−11	3.02E−11	2.57E−07	2.23E−09	3.02E−11	1
	Std	7.91E+02	1.03E+03	3.21E+03	2.54E+03	8.53E+02	2.12E+03
F23	Max	4.09E+03	3.92E+03	5.09E+03	3.94E+03	4.05E+03	3.40E+03
	Mean	3.86E+03	3.76E+03	4.74E+03	3.78E+03	3.92E+03	3.24E+03
	Min	3.74E+03	3.63E+03	4.19E+03	3.57E+03	3.72E+03	3.11E+03
	*p*-value	3.02E−11	3.02E−11	3.02E−11	3.02E−11	3.02E−11	1
	Std	7.61E+01	7.42E+01	2.86E+02	9.61E+01	7.94E+01	7.71E+01
F24	Max	4.81E+03	4.52E+03	8.21E+03	4.73E+03	4.70E+03	4.14E+03
	Mean	4.64E+03	4.31E+03	5.83E+03	4.51E+03	4.54E+03	3.80E+03
	Min	4.36E+03	4.17E+03	4.65E+03	4.29E+03	4.39E+03	3.64E+03
	*p*-value	3.02E−11	3.02E−11	3.02E−11	3.02E−11	3.02E−11	1
	Std	1.14E+02	7.48E+01	6.72E+02	1.19E+02	7.63E+01	1.13E+02
F25	Max	9.07E+03	8.57E+03	6.61E+03	6.47E+03	4.78E+03	3.80E+03
	Mean	7.21E+03	6.32E+03	4.61E+03	5.43E+03	4.28E+03	3.63E+03
	Min	5.49E+03	5.12E+03	3.97E+03	4.67E+03	3.98E+03	3.42E+03
	*p*-value	3.02E−11	3.02E−11	3.02E−11	3.02E−11	3.02E−11	1
	Std	8.15E+02	7.75E+02	6.29E+02	4.52E+02	1.97E+02	8.46E+01
F26	Max	2.48E+04	1.85E+04	3.06E+04	2.15E+04	2.21E+04	1.28E+04
	Mean	2.03E+04	1.68E+04	2.06E+04	1.93E+04	1.90E+04	1.06E+04
	Min	1.50E+04	1.52E+04	1.57E+04	1.69E+04	1.68E+04	9.25E+03
	*p*-value	3.02E−11	3.02E−11	3.02E−11	3.02E−11	3.02E−11	1
	Std	2.09E+03	8.31E+02	3.40E+03	1.35E+03	1.37E+03	8.56E+02
F27	Max	4.99E+03	4.15E+03	4.76E+03	5.00E+03	4.13E+03	3.87E+03
	Mean	4.68E+03	3.92E+03	4.04E+03	4.33E+03	3.90E+03	3.61E+03
	Min	4.35E+03	3.71E+03	3.73E+03	3.96E+03	3.74E+03	3.46E+03
	*p*-value	3.02E−11	1.78E−10	2.37E−10	3.02E−11	5.57E−10	1
	Std	1.78E+02	1.02E+02	2.48E+02	2.08E+02	1.15E+02	9.25E+01
F28	Max	1.15E+04	1.38E+04	1.01E+04	9.64E+03	6.70E+03	4.44E+03
	Mean	9.33E+03	1.04E+04	6.19E+03	7.28E+03	5.13E+03	3.93E+03
	Min	7.63E+03	6.25E+03	3.90E+03	4.96E+03	4.19E+03	3.62E+03
	*p*-value	3.02E−11	3.02E−11	8.10E−10	3.02E−11	1.21E−10	1
	Std	1.08E+03	1.91E+03	1.88E+03	8.69E+02	5.50E+02	2.14E+02
F29	Max	9.51E+03	1.06E+04	9.02E+03	1.06E+04	1.10E+04	8.38E+03
	Mean	8.81E+03	9.44E+03	8.03E+03	8.73E+03	9.95E+03	7.16E+03
	Min	7.23E+03	7.62E+03	6.43E+03	7.65E+03	8.36E+03	5.48E+03
	*p*-value	1.78E−10	5.49E−11	5.86E−06	1.33E−10	3.34E−11	1
	Std	5.11E+02	6.52E+02	6.56E+02	6.82E+02	7.03E+02	5.72E+02
F30	Max	1.04E+08	1.83E+07	3.46E+09	2.73E+07	5.67E+06	1.25E+05
	Mean	4.52E+07	4.12E+06	1.02E+09	1.07E+07	1.83E+06	6.02E+04
	Min	1.75E+07	8.33E+05	4.72E+06	3.51E+06	5.53E+05	2.10E+04
	*p*-value	3.02E−11	3.02E−11	3.02E−11	3.02E−11	3.02E−11	1
	Std	2.30E+07	3.66E+06	9.97E+08	6.24E+06	1.22E+06	2.12E+04
